# Discovery of H_2_ Receptor Antagonists as Colistin Enhancers by Targeting Acid Stress Response

**DOI:** 10.1002/advs.202514165

**Published:** 2026-02-25

**Authors:** Jinju Cai, Mengping He, Jianya Luo, Shuang Zhou, Tengyue Zhang, Jianing Li, Zhiqiang Wang, Yuan Liu

**Affiliations:** ^1^ Jiangsu Co‐innovation Center For Prevention and Control of Important Animal Infectious Diseases and Zoonoses College of Veterinary Medicine Yangzhou University Yangzhou China; ^2^ Joint International Research Laboratory of Agriculture and Agri‐Product Safety the Ministry of Education of China Yangzhou University Yangzhou China; ^3^ Institute of Comparative Medicine Yangzhou University Yangzhou China

**Keywords:** antibiotic adjuvant, colistin, gram‐negative bacteria, resistance, YqgB

## Abstract

Plasmid‐mediated colistin resistance gene *mcr* has markedly diminished the effectiveness of colistin in managing multidrug‐resistant (MDR) Gram‐negative bacterial infections. Employing antibiotic adjuvants to revive colistin susceptibility offers a valuable solution to address this issue. Intestinal pathogens have evolved sophisticated acid resistance systems to withstand the acidic environment, a key adaptation that facilitates subsequent infection. Utilizing transposon sequencing, here we identify *yqgB* gene as a crucial target for restoring colistin susceptibility in *mcr*‐positive bacteria, particularly under acidic conditions. *yqgB* deficiency drastically enhances colistin's bactericidal effect, which is attributed to the alteration of phospholipid composition and reduction in lipopolysaccharide modification. Using deep learning‐based screening, we find that H_2_ receptor antagonists (ebrotidine, ranitidine and famotidine) can act as dual inhibitors of MCR and YqgB proteins, thus exhibiting excellent synergistic activity with colistin against *mcr*‐positive bacteria under both neutral and acidic conditions. This combination not only bolsters colistin's efficacy but also impedes the evolution and spread of colistin resistance. In animal models infected with *mcr*‐positive pathogens, the co‐administration of ranitidine and colistin demonstrates superior therapeutic outcomes against both systemic and intestinal infections. Our findings highlight the potential of targeting the bacterial acid stress response system to counteract *mcr*‐mediated colistin resistance and offer a promising therapeutic strategy for the treatment of MDR bacterial infections.

## Graphical abstract

This study identifies YqgB as a key target for restoring colistin susceptibility in *mcr*‐positive pathogens under acidic conditions by remodeling phospholipid composition and reducing LPS modification. Deep learning‐based screening reveals H_2_ receptor antagonists as novel colistin adjuvants. Further investigations indicate that ranitidine and famotidine effectively improve therapeutic outcomes of colistin in animal models of infection.

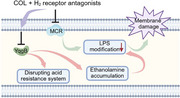

## 1 Introduction

The advent and clinical deployment of antibiotics represent a pivotal milestone in the annals of human history and modern medicine. These agents have revolutionized medical practice and remain indispensable in the arsenal of life‐saving treatments [1]. However, the proliferation of drug‐resistant pathogens, notably the emergence of superbugs, poses a significant threat to global public health [2, 3]. It is estimated that by 2050, antibiotic‐resistant bacteria will cause 10 million deaths annually [4]. Colistin is typically the last line of defense for treating multidrug‐resistant (MDR) Gram‐negative pathogens [5]. Its positively charged residues interact with the negatively charged lipid A component of lipopolysaccharides (LPS), disrupting membrane integrity and exerting bactericidal effects [6]. The MCR‐1 enzyme [7], a novel member of the phosphoethanolamine (pEtN) transferase family, modifies lipid A by transferring pEtN residues, thereby reducing its negative charge and diminishing the antibacterial activity of colistin [8]. Biochemical and structural investigations have substantially advanced the understanding of MCR‐mediated colistin resistance [9, 10, 11]. The *mcr‐1* gene and its variants, carried on plasmids, have disseminated globally through horizontal gene transfer, contributing to the rise of MDR superbugs [12, 13, 14, 15].

To address the urgent need for novel therapeutics against *mcr*‐harboring multidrug‐resistant (MDR) Gram‐negative bacteria, two primary strategies have emerged: the discovery of new antibiotics, exemplified by darobactin from *Photorhabdus* isolates [16], and the exploration of antibiotic analogs, such as macolacin [17], which shows enhanced activity against *mcr*‐positive bacteria. However, the development of these new agents is lagging due to high costs, lengthy development periods, and limited profitability [18]. An economically viable alternative is the use of antibiotic adjuvants [19, 20], with clavulanic acid as a successful case, enhancing the efficacy of penicillins and cephalosporins by inhibiting β‐lactamases [21]. Our previous studies have identified both melatonin and sodium caprate as potential colistin adjuvants [22, 23], with synergistic mechanisms primarily associated with the reduction of MCR expression and/or function. Current colistin synergists are predominantly directed against MCR enzymes, and their activity may be compromised by mutations in resistance enzymes or analogous LPS modification mechanisms. Identifying key sensitization genes or pathways for colistin synergism offers a more valuable target for enhancing colistin's efficacy against MDR bacteria. For instance, a recent study demonstrated that inhibiting bacterial fatty acid synthesis can deplete lipid synthesis, thereby restoring colistin susceptibility [24].

Enteric pathogens like *Escherichia coli* and *Salmonella* can colonize the gut and cause disease. They encounter a less acidic pH (4‐6) in the small intestine due to organic acids from the normal flora [25]. These pathogens have developed multiple acid resistance (AR) systems to cope with acid stress, including AR1‐AR5. AR1 is regulated by the alternative σ factor (RpoS) and cAMP receptor protein (CRP) [26, 27], while AR2‐AR5 depend on specific amino acids and confer resistance by decarboxylation, maintaining internal pH [28, 29]. These systems are crucial for bacterial pathogenicity and survival in acidic environments, but their link to antibiotic susceptibility remains poorly understood.

**FIGURE 1:**
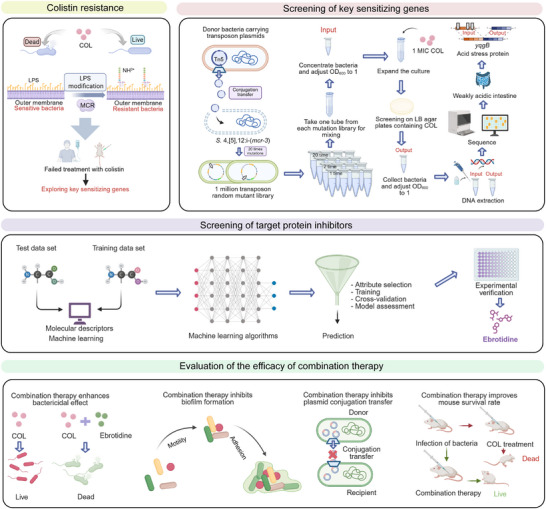
Schematic illustration of screening novel colistin enhancers by targeting key sensitizing genes of *mcr‐*positive bacteria.

In this study, we endeavored to identify key targets for restoring colistin susceptibility in MCR‐positive bacteria and elucidate the underlying sensitization mechanisms (Figure 1). Notably, we discovered that targeting the acid stress response system can overcome MCR‐mediated colistin resistance by altering phospholipid composition and reducing LPS modification. Moreover, we employed computer‐aided virtual screening to identify potential dual inhibitors of MCR and YqgB proteins, and subsequently assessed their synergistic activity with colistin against MCR‐carrying bacteria. Excitingly, our findings indicate that H_2_ receptor antagonists, such as ebrotidine, ranitidine, and famotidine, act as novel colistin enhancers, demonstrating the potential to significantly potentiate colistin's effectiveness both in vitro and in murine models.

## 2 Results

### 2.1 YqgB Is Responsible for the Improved Colistin Susceptibility of *Mcr*‐Positive Bacteria

**FIGURE 2:**
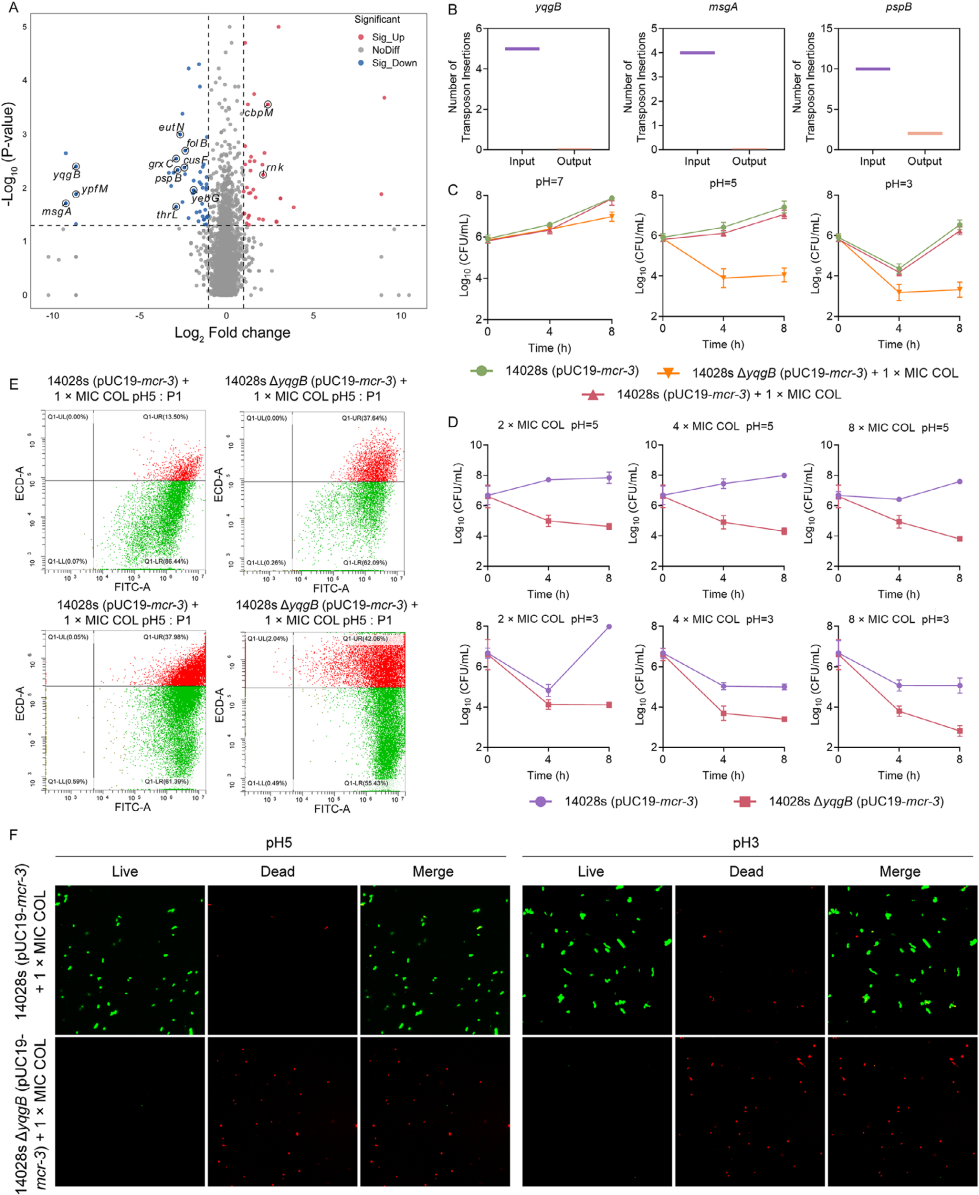
*yqgB* deficiency restores colistin susceptibility in *mcr*‐positive bacteria.(A) Changes in the number of gene insertion sites in transposon sequencing results. (B) The number of transposon insertion sites for *yqgB*, *msgA*, and *pspB* genes in the input and output libraries. (C) Effect of *yqgB* deficiency on the bactericidal effect of colistin (COL) in different pH environments. (D) Time‐dependent killing curves of STm 14028s (pUC19‐*mcr‐3*) with or without *yqgB* gene treated with various concentrations of colistin (2 × MIC, 4 × MIC, 8 × MIC) in the acidic environment. (E) LIVE/DEAD *Bac*Light viability assay of STm 14028s (pUC19‐*mcr‐3*) and the *yqgB* deficient strains after exposure to 1 × MIC colistin, determined by flow cytometry analysis. (F) Confocal laser scanning microscope (CLSM) of the living and dead status of the STm 14028s (pUC19‐*mcr‐3*) with *yqgB* gene deletion after treatment with 1 × MIC colistin under acidic environment. Data were from three independent experiments and were presented as mean ± SD.

To identify genetic determinants that resensitize *mcr*‐positive bacteria to colistin, we conducted transposon‐directed insertion‐site sequencing (TraDIS). Genomic DNA from input and output libraries yielded ∼70 million reads per sample, 89% of which carried transposon sequences and mapped uniquely to the reference genome. We recovered 369,901 insertions in the input library and 311,528 in the output library. Comparison of insertion abundance before and after exposure to 1 × MIC colistin identified genes whose representation collapsed under antibiotic pressure; these genes are indispensable for survival in the presence of colistin. Among them, *yqgB*, *msgA* and *pspB* exhibited the most pronounced depletion and were selected for further validation (Figure 2A,B). We constructed gene knockout strains in STm 14028s (pUC19‐*mcr‐3*) and assessed their colistin susceptibility using time‐killing curves. We found that the deletion of *pspB* and *msgA* genes did not affect the antibacterial activity of colistin against STm 14028s (pUC19‐*mcr‐3*) (Figure S1). Notably, the deletion of *yqgB* in STm 14028s (pUC19‐*mcr‐3*) modestly enhanced the bactericidal effect of colistin, reducing bacterial counts by approximately 1‐log10 at pH 7.0 (Figure 2C). Correspondingly, the MIC of colistin for STm 14028s Δ*yqgB* (pUC19‐*mcr‐3*) was reduced (2 µg/mL) compared to the wild‐type (4 µg/mL).

Given that Enterobacteriaceae have developed acid resistance mechanisms essential for survival in the stomach and intestine [30], and considering YqgB's role in acid stress response [31], we investigated the impact of *yqgB* deletion on colistin's efficacy in acidic conditions. At pH 5 and pH 3, the absence of *yqgB* significantly potentiated the bactericidal action of 1 × MIC colistin, which was minimally effective against STm 14028s (pUC19‐*mcr‐3*) (Figure 2C). This effect was more pronounced with higher colistin concentrations (2 × MIC, 4 × MIC, and 8 × MIC) (Figure 2D). Flow cytometry, using SYTO 9 and PI to stain live and dead cells, revealed a significant increase in dead cells (red fluorescence) in STm 14028s Δ*yqgB* (pUC19‐*mcr‐3*) following 1 × MIC colistin treatment at pH 5 and pH 3, compared to the wild‐type (Figure 2E). Confocal laser scanning microscopy (CLSM) confirmed that colistin was more effective against STm 14028s Δ*yqgB* (pUC19‐*mcr‐3*) than the wild‐type strain at pH 5 and pH 3 environments (Figure 2F). Collectively, these findings indicate that *yqgB* deletion in *mcr*‐positive bacteria enhances colistin's bactericidal activity, particularly under acidic conditions.

### 2.2 Deletion of *yqgB* Inhibits the Arginine‐Dependent Acid Resistance System

**FIGURE 3:**
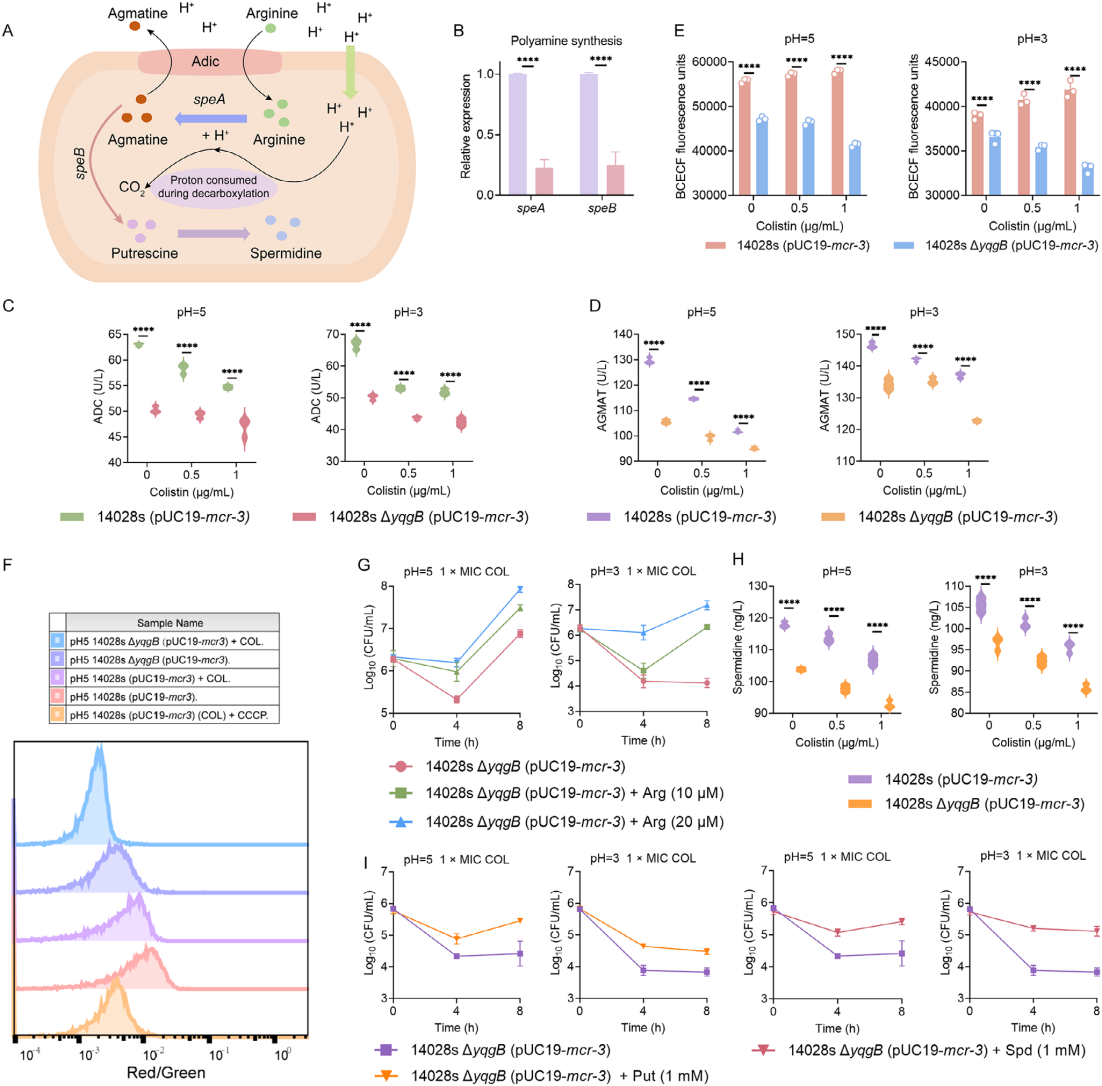
yqgB deficiency inhibits the arginine‐dependent acid resistance system.(A) Schematic illustration of the involvement of *speA* and *speB* genes in the arginine‐dependent acid resistance system and polyamine synthesis. (B) RT‐qPCR analysis of the expression of *speA* and *speB* genes. (C, D) Detection of arginine decarboxylase (ADC) and agmatine ureohydrolase (AGMAT) content. (E) The intracellular H^+^ concentration of STm 14028s (pUC19‐*mcr‐3*) and its *yqgB* deficient strains under the action of colistin, detected using a pH indicator BCECF‐AM. (F) Proton motive force (PMF) measurement by flow cytometry analysis. CCCP was used as the positive control. (G) Time‐dependent killing curve of *yqgB*‐deleted strain exposed to colistin in the presence of arginine (Arg) under acidic environment. (H) Detection of polyamine content in STm 14028s (pUC19‐*mcr‐3*) and its *yqgB* deficient strains treated with colistin in acidic environment. (I) Effect of the addition of putrescine (Put) and spermidine (Spd) on the bactericidal activity of colistin against STm 14028s Δ*yqgB* (pUC19‐*mcr‐3*) in an acidic environment. Data were from three independent experiments and were presented as mean ± SD. Two‐way ANOVA was used to determine the statistical significance (****p < 0.0001).

Next, we explored the relationship between *yqgB* gene deletion and acid resistance in *mcr*‐positive bacteria. Related articles have shown that the ability of *yqgB* to exert acid resistance is dependent on the formation of polyamines [31]. The biosynthesis pathway of polyamines is shown in Figure 3A. In an acidic environment, arginine is converted into agmatine by arginine decarboxylase, which consumes H^+^. Subsequently, agmatine is converted into putrescine under the action of agmatine ureohydrolase. Under the action of spermidine synthase, putrescine forms spermidine. Polyamines block porins and decrease membrane permeability, activities that may protect cells in acid [32]. RT‐qPCR analysis revealed that *yqgB* deletion downregulated *speA* and *speB* (Figure 3B), which encode arginine decarboxylase (ADC) and agmatine ureohydrolase (AGMAT), respectively. These enzymes constitute a metabolic pathway converting arginine to spermidine [33]. Measurements of ADC and AGMAT levels in STm 14028s Δ*yqgB* (pUC19‐*mcr‐3*) showed a decrease at pH 5 and pH 3 compared with the wild‐type strain (Figure 3C,D). Since arginine consumes H^+^ during the formation of agmatine by ADC, we hypothesized that reduced ADC expression could lead to increased intracellular H^+^ and diminished proton motive force (PMF). To test this, we used a fluorescent probe BCECF‐AM to detect intracellular H^+^ in the acidic environment at different concentrations of colistin. A lower fluorescence value corresponds to a higher concentration of intracellular H^+^. Consequently, we detected higher intracellular H^+^ levels in STm 14028s Δ*yqgB* (pUC19‐*mcr‐3*) at pH 5 and pH 3 (Figure 3E). Furthermore, PMF was reduced in STm 14028s Δ*yqgB* (pUC19‐*mcr‐3*) at pH 5, irrespective of colistin addition (Figure 3F). The addition of arginine mitigated the bactericidal effect of colistin on STm 14028s Δ*yqgB* (pUC19‐*mcr‐3*) under acidic conditions (Figure 3G). Consistent with gene expression findings, spermidine content was reduced in the absence of *yqgB* at low pH (Figure 3H). Supplementation with putrescine and spermidine at pH 5 and pH 3 diminished colistin's bactericidal activity against STm 14028s Δ*yqgB* (pUC19‐*mcr‐3*) (Figure 3I). Collectively, these data indicate that *yqgB* deletion disrupts bacterial acid resistance by inhibiting the arginine‐dependent acid resistance system and polyamine synthesis.

### 2.3 Deletion of *yqgB* Alters Phospholipid Composition and Reduces LPS Modification

**FIGURE 4:**
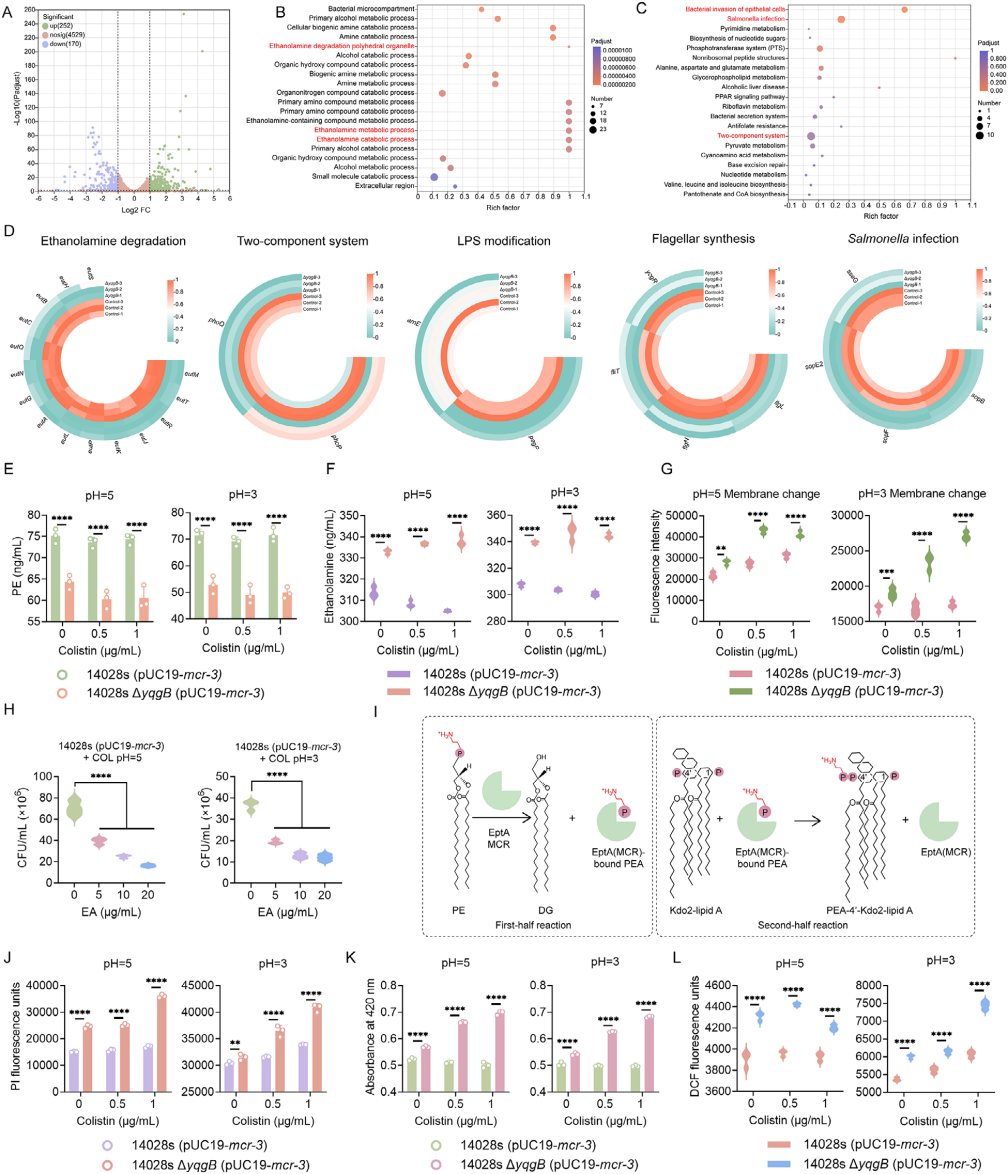
*yqgB* deficiency alters phospholipid composition and reduces LPS modification. (A‐C) Volcano plot (A), GO (B) and KEGG enrichment (C) analysis of the differential expression genes (DEGs) in the STm 14028s (pUC19‐*mcr‐3*) and its *yqgB*‐deficient strain. The x and y axes in A represent the expression changes and corresponding statistically significant degree, respectively. (D) Selected DEGs participating in multiple pathways, including ethanolamine degradation system, two‐component system, LPS modification, flagellar synthesis and *Salmonella* infection. (E and F) Effect of *yqgB* deficiency on the content of PE (E) and ethanolamine (F) in *mcr*‐positive bacteria treated with colistin under acidic environment. (G) Cell membrane surface charge of *mcr*‐positive bacteria with *yqgB*‐deficient strain under acidic environment. (H) Effect of ethanolamine addition on the activity of colistin against STm 14028s (pUC19‐*mcr‐3*). (I) Schematic diagram of MCR protein transferring phosphoethanolamine to LPS. (J) Membrane permeability of *mcr‐*positive bacteria with *yqgB* deficiency under treatment with colistin, measured using propidium iodide (PI). (K) The activity of extracellular β‐galactosidase in *mcr*‐positive bacteria with *yqgB* gene deletion exposed to different concentrations of colistin. (L) ROS levels of STm 14028s (pUC19‐*mcr‐3*) and its *yqgB*‐deficient strain under treatment with colistin in an acidic environment. Data were shown as mean ± SD, and two‐way or one‐way ANOVA was used to evaluate statistical significance (**p < 0.01, ***p < 0.001, and ****p < 0.0001).

To investigate the molecular mechanisms underlying the enhanced colistin susceptibility of *yqgB*‐deficient strain in acidic conditions, we performed transcriptome analysis. Comparative RNA sequencing between STm 14028s (pUC19‐*mcr‐3*) and its *yqgB*‐deficient counterpart revealed that 252 genes were upregulated and 170 genes were downregulated in the absence of *yqgB* (Figure 4A). Gene Ontology (GO) and KEGG pathway analyses highlighted that downregulated genes were enriched in pathways such as ethanolamine degradation, *Salmonella* infection, and bacterial invasion of epithelial cells (Figure 4B,C). Additionally, *yqgB* deletion downregulated genes involved in fatty acid biosynthesis, two‐component systems, LPS modification, and flagellar synthesis (Figure 4D; Figure S2). Notably, the two‐component system PhoP/Q, known to stabilize LPS and outer membrane integrity under acid stress by upregulating PagP [34], was downregulated in the *yqgB*‐deficient strain. PagP facilitates the transfer of acyl chains to lipid A, forming a more hepta‐acylated lipid A [35], which is crucial for membrane homeostasis. The transcriptome data suggested that *yqgB* deficiency led to a downregulation of *pagP*, potentially disrupting membrane stability. Furthermore, the downregulation of fatty acid synthesis in the Δ*yqgB* strain altered phospholipid composition, reducing phosphatidylethanolamine (PE) levels (Figure 4E), which is associated with colistin susceptibility [24].

Ethanolamine degradation was also downregulated upon *yqgB* deletion, as confirmed by increased ethanolamine levels in the *yqgB*‐deficient strain (Figure 4F). Ethanolamine, an analog of pEtN, can competitively inhibit MCR protein, restricting pEtN incorporation into lipid A of LPS [36], thereby reducing the cell membrane's positive charge. Consistently, the *yqgB*‐deficient strain exhibited a significant decrease in surface charge (Figure 4G), suggesting that the deletion of *yqgB* gene reduced LPS modification by inhibiting ethanolamine degradation. Moreover, the addition of ethanolamine enhanced the bactericidal effect of colistin against STm 14028s (pUC19‐*mcr‐3*) in a dose‐dependent manner (Figure 4H).

MCR, a phosphoethanolamine (pEtN) transferase, modifies lipid A in LPS, reducing its negative charge and affinity for colistin, thus conferring resistance (Figure 4I). We hypothesized that the altered phospholipid composition and reduced LPS modification due to *yqgB* deletion could restore colistin's action on the cell mefmbrane. This was confirmed by increased membrane permeability in the *yqgB*‐deficient strain under colistin treatment, as measured by propidium iodide uptake and β‐galactosidase activity (Figure 4J,K). Membrane damage is often associated with reactive oxygen species (ROS) production, which plays a critical role in antibiotic‐mediated bacterial killing. Indeed, *yqgB* knockout bacteria showed increased ROS generation under colistin treatment in acidic conditions (Figure 4L).

**FIGURE 5:**
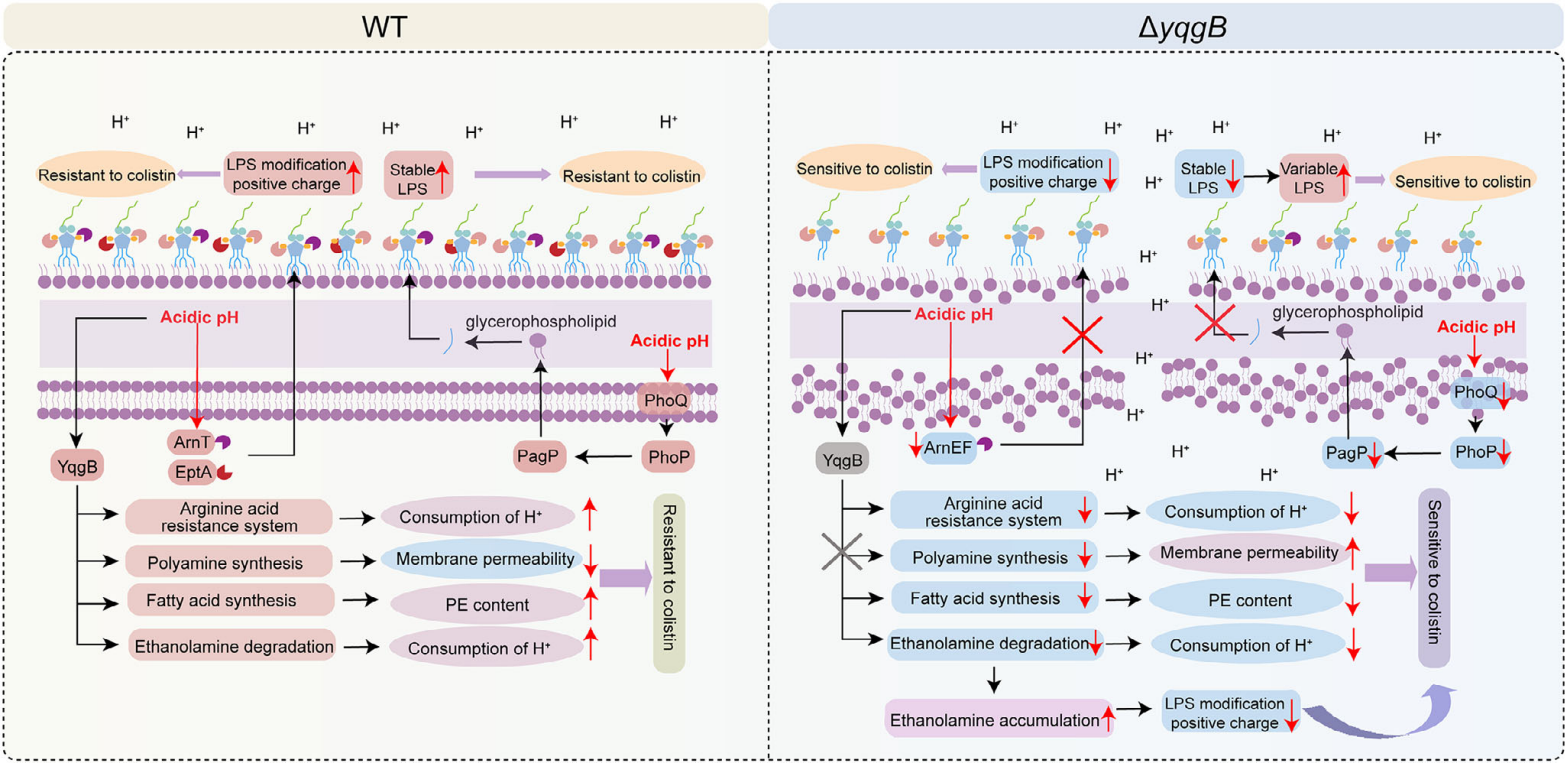
Schematic illustration of the mechanism by which *yqgB* deficiency restores the susceptibility of *mcr*‐positive bacteria to colistin.Schematic illustration of the mechanism by which the deletion of *yqgB* gene in *mcr*‐positive bacteria enhances the bactericidal effect of colistin in an acidic environment.

Together, our results indicate that the *yqgB* deletion remodels phospholipid composition and reduces LPS modification, which enhances membrane permeability and ROS generation in response to colistin, ultimately rendering *mcr*‐positive bacteria more susceptible to colistin treatment (Figure 5) (Figure S3).

### 2.4 Deep Learning‐Driven the Discovery of Novel Colistin Enhancers

**FIGURE 6:**
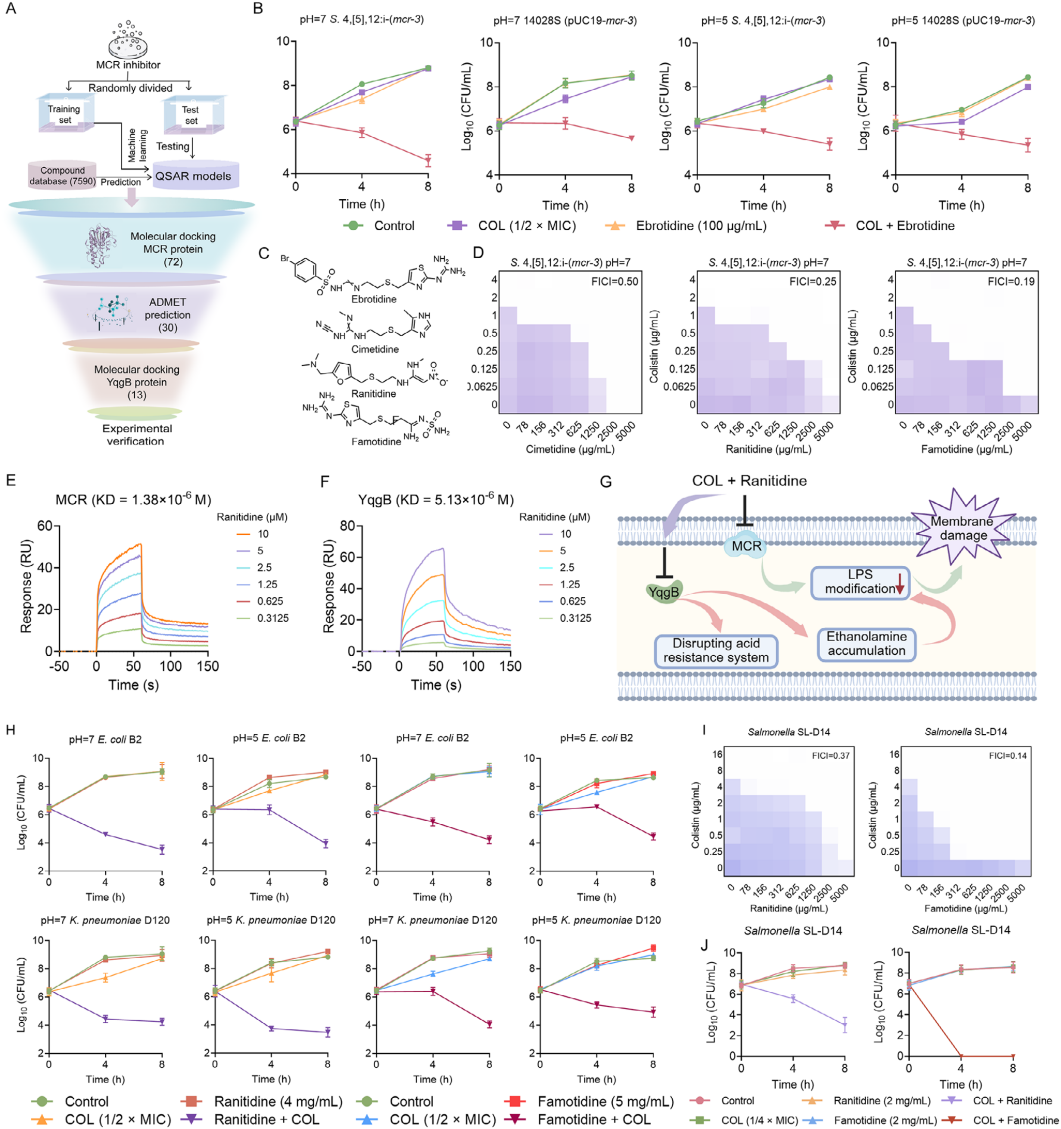
Machine learning‐driven discovery of H_2_ receptor antagonists as novel colistin enhancers.(A) Schematic illustration of machine learning‐based screening of novel colistin enhancers targeting both MCR and YqgB proteins. (B) Time‐dependent killing curves of *mcr‐*positive bacteria treated with colistin and ebrotidine alone or their combination under neutral and acidic conditions. (C) Chemical structures of ebrotidine analogues. (D) Checkerboard broth microdilution assays between cimetidine, ranitidine, famotidine and colistin against *mcr*‐positive *S*. 4,[5],12:i‐ (*mcr‐3*). (E) SPR analysis of ranitidine and MCR protein. (F) SPR analysis of ranitidine and YqgB protein. (G) Schematic diagram of ranitidine targeting MCR protein and YqgB protein to enhance the bactericidal effect of colistin. (H) Time‐dependent killing curves of *E. coli* B2 (*mcr‐1*) and *K. pneumoniae* D120 (*mcr‐8*) treated with colistin and ranitidine/famotidine alone or their combination under neutral and acidic environment. (I) Checkerboard assay of ranitidine/famotidine combined with colistin against *Salmonella* SL‐D14. (J) Analysis of the bactericidal curve of ranitidine/famotidine combined with colistin against *Salmonella* SL‐D14. Experiments were carried out with three biological replicates and all data were given as mean ± SD.

Having shown that *yqgB* deficiency enhances colistin susceptibility in *mcr*‐positive bacteria under acidic conditions, we sought to identify a dual‐target inhibitor that could disrupt both MCR function and the acid resistance system, thereby restoring colistin activity under host‐like conditions (Figure 6A). A quantitative structure‐activity relationship (QSAR) model was first constructed by partitioning known MCR inhibitors into training and test sets; the model exhibited robust predictive accuracy. Among 7,590 compounds subsequently screened in silico, 1,052 were predicted to inhibit MCR (Figure S4A). Structure‐based docking retained 213 molecules, 72 of which displayed high binding affinity (ΔG_bind < −7.0 kcal/mol) to the MCR active site (Figure S4B). After filtering for absorption, distribution, metabolism, excretion and toxicity (ADMET) properties, 30 compounds advanced to docking against YqgB, yielding 13 dual‐target candidates (Figure S4C,D). MIC profiling revealed that only ebrotidine, an H_2_‐receptor antagonist with gastroprotective properties [37], significantly potentiated colistin's activity at both pH 7 and pH 5 (Tables S1,S2).

Next, we performed time‐dependent killing curves of *mcr*‐positive STm 14028s (pUC19‐*mcr‐3*) and *S*. 4,[5],12:i‐ (*mcr‐3*) under colistin alone or in combination with ebrotidine. The results showed that under pH 7 and pH 5 conditions, colistin and ebrotidine alone at subinhibitory concentrations had no direct bactericidal effect, but their combination resulted in a reduction of CFUs above 4‐log10 (Figure 6B). Moreover, we tested the synergistic activity of three additional widely available H_2_ receptor antagonists (cimetidine, ranitidine, and famotidine) with colistin (Figure 6C). The results showed that two compounds, including ranitidine and famotidine, displayed excellent synergistic activity with colistin against *mcr*‐positive bacteria (Figure 6D). In addition, this synergistic activity was not observed in *mcr*‐negative bacteria (Figure S5), further supporting our idea that ranitidine and famotidine are potential inhibitors of MCR protein.

Molecular docking revealed that ebrotidine (ΔG_bind = −7.5 kcal/mol), ranitidine (−7.8 kcal/mol) and famotidine (−7.3 kcal/mol) occupy the MCR active site, whereas cimetidine (−2.5 kcal/mol) lacks measurable affinity (Figure S6). The same three ligands also docked into the conserved pocket of YqgB (ebrotidine −7.0 kcal/mol, ranitidine −7.9 kcal/mol, famotidine −7.7 kcal/mol; cimetidine −2.7 kcal/mol) (Figure S7). Furthermore, we conducted surface plasmon resonance (SPR) analyses to experimentally validate the predicted interactions between ranitidine and the target proteins. The results showed that ranitidine directly interacted with both the MCR protein and the YqgB protein, exhibiting strong binding affinities. The equilibrium dissociation constants (KD) were 1.38 × 10^−6^ m for MCR (Figure 6E) and 5.13 × 10^−6 ^M for YqgB (Figure 6F), respectively. These results demonstrated that ebrotidine, ranitidine and famotidine act as dual MCR/YqgB inhibitors, potentiating colistin under both neutral and acidic conditions (Figure 6G).

Given the clinical prevalence of ranitidine and famotidine, we selected these compounds for further investigation. We assessed their synergistic activity against other *mcr*‐positive bacteria, including *E. coli* B2 (*mcr‐1*) and *K. pneumoniae* D120 (*mcr‐8*). Ranitidine and famotidine significantly enhanced colistin's bactericidal activity against *mcr*‐carrying *E. coli* and *K. pneumoniae* in both neutral and weakly acidic conditions (Figure 6H). Beyond plasmid‐borne *mcr*, chromosomal mutations in *pmrAB*, *phoPQ* or their repressor *mgrB* constitutively remodel LPS and surface charge, yielding high‐level colistin resistance by drastically reducing drug binding [38]. Checkerboard assays revealed synergistic activity (FICI ≤ 0.5) for colistin plus ranitidine or famotidine against the chromosomally resistant *Salmonella* SL‐D14 (Figure 6I). Time‐dependent killing curves showed complete eradication within 4 h with the famotidine‐colistin combination (Figure 6J), and flow cytometry confirmed a significantly higher proportion of dead cells relative to either agent alone (Figure S8).

Studies have shown that the formation of bacterial biofilms can reduce the therapeutic efficacy of antibiotics [39, 40]. We examined the impact of *yqgB* gene deletion on biofilm formation and found that it was significantly inhibited in *yqgB*‐deficient strains at pH 5 and pH 3 (Figure S9A). We then investigated whether ranitidine and famotidine could enhance colistin's inhibitory effect on biofilm formation and the eradication of mature biofilms under neutral and weakly acidic conditions. The drug combination, despite lacking direct bactericidal activity at low concentrations, significantly inhibited biofilm formation of *mcr*‐positive bacteria (Figure S9B). Additionally, ranitidine and famotidine enhanced the eradication of mature biofilms by colistin (Figure S9C). Collectively, our results indicate that H_2_ receptor antagonists effectively enhance colistin's activity against *mcr*‐positive bacteria under both neutral and weakly acidic conditions.

### 2.5 Ranitidine and Famotidine Suppress the Evolution and Spread of Colistin Resistance

**FIGURE 7:**
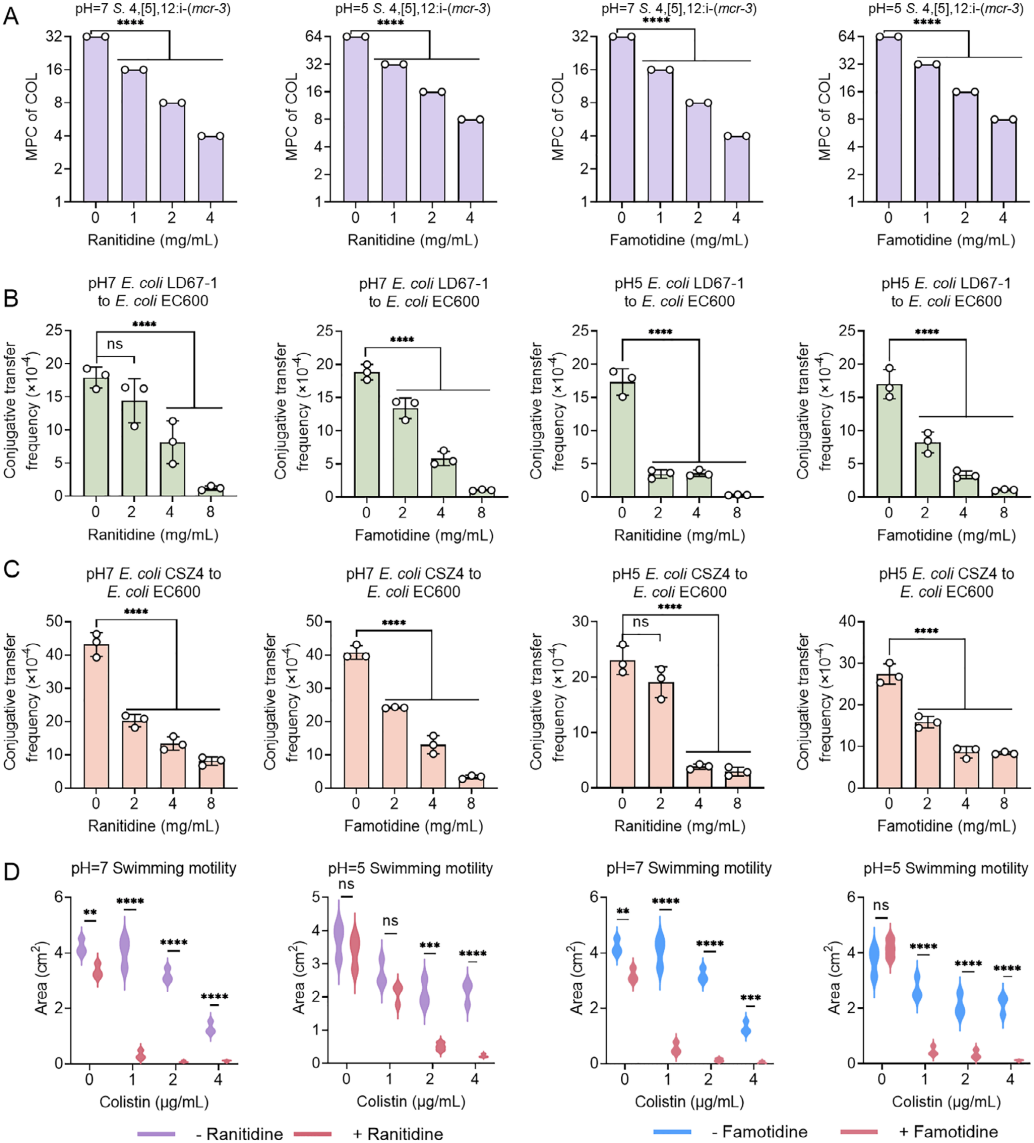
Ranitidine and famotidine suppress the evolution and spread of colistin resistance.(A) MPC values of colistin in the presence of increasing concentrations of ranitidine or famotidine against *S*. 4,[5],12:i‐ (*mcr‐3*) under neutral and acidic conditions. (B, C) Conjugative transfer frequency of *mcr‐1*‐carrying plasmids from two clinical isolates (*E. coli* LD67‐1 and *E. coli* CSZ4) to the recipient bacteria *E. coli* EC600 in the presence of ranitidine or famotidine under neutral and acidic conditions. (D) Swimming motility assay of *S*. 4,[5],12:i‐ (*mcr‐3*) after treatment with colistin alone or in combination with ranitidine/famotidine under neutral and acidic environment. Experiments were carried out with three biological replicates and all data were presented as mean ± SD. One‐way or two‐way ANOVA was used to determine the statistical significance (ns, not significant, **p < 0.01, ***p < 0.001, ****p < 0.0001).

To assess the potential of ranitidine and famotidine in preventing the evolution of colistin resistance, we conducted mutation prevention concentration (MPC) assays. We observed that the addition of these H_2_ receptor antagonists under neutral and weakly acidic conditions led to a dose‐dependent decrease in the MPC values of colistin against drug‐resistant bacteria (Figure 7A). Given the widespread dissemination of the *mcr‐1* gene through conjugative plasmids, we further investigated the impact of ranitidine and famotidine on the conjugation frequency of *mcr*‐harboring plasmids. Strikingly, both compounds significantly reduced the conjugative transfer frequency of *mcr‐1*‐carrying plasmids under neutral and weakly acidic conditions (Figure 7B,C). Additionally, our results showed that ranitidine and famotidine significantly decreased the heteroresistance frequency (HRF) of the *K. pneumoniae* YZ6 (Figure S10), underscoring their capacity to curb the emergence of resistant subpopulations that drive therapeutic failure and relapse [41]. These findings suggest that ranitidine and famotidine can impede the evolution and spread of colistin resistance.

Transcriptomic analysis revealed that deletion of the *yqgB* gene led to a marked suppression of the *Salmonella* infection pathway. RT‐qPCR analysis verified the down‐regulation of SPI‐1 genes (*sopB, sopE2, sipA*) and SPI‐2 genes (*sseG, sseF*) in the Δ*yqgB* mutant (Figure S11A). Consistently, mice challenged with the mutant exhibited significantly lower bacterial burdens in the liver, cecum, colon and feces than the original strain (Figure S11B). The bacterial flagellum, an intricate molecular apparatus, is essential for swimming motility and contributes significantly to the pathogenicity of bacteria. Transcriptomic data indicated that the deletion of the *yqgB* gene resulted in the downregulation of genes associated with flagellar biosynthesis. Consequently, we explored the impact of ranitidine and famotidine, when combined with colistin, on bacterial motility. As shown in Figure 7D, their combination markedly reduced bacterial motility, as evidenced by a notable reduction in the motility area (Figure S12). These findings highlight the potential of ranitidine and famotidine to inhibit bacterial virulence by targeting YqgB.

### 2.6 Restoration of Therapeutic Efficacy of Colistin by Ranitidine In Vivo

Given that ranitidine can significantly enhance the bactericidal effect of colistin against *mcr*‐positive bacteria in vitro, we next sought to evaluate the therapeutic potential of their combination in vivo. Prior to this, we evaluated the safety of ranitidine combined with colistin. The hemolytic activity test of red blood cells (RBCs) showed that no hemolysis was detected under this combination treatment (Figure S13). Subsequently, we assessed the toxicity profile of this combination in mice via intraperitoneal injection and observed no significant discrepancies in hematological parameters or serum biochemistry indices between the treatment and control groups (Tables S4 and S5).

**FIGURE 8:**
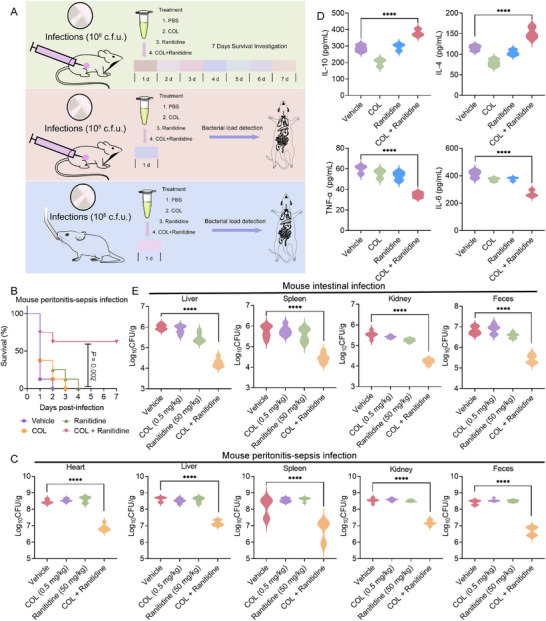
Ranitidine rescues colistin activity in animal models of infection.(A) Schematic illustration of experimental protocols for two animal models. (B) Survival rate of mice infected by *S*. 4,[5],12:i‐ (*mcr‐3*) during 7 days post‐infection. ICR mice (n = 8 per group) were given a lethal dose of *S*. 4,[5],12:i‐ (*mcr‐3*) (6 × 10^8^ CFUs), and treated with a single dose of colistin (0.5 mg/kg), ranitidine (50 mg/kg), a combination of colistin plus ranitidine (0.5 + 50 mg/kg). *P* values were determined by log‐rank (Mantel‐Cox) test. (C) Bacterial loads in the mouse organs (heart, liver, spleen and kidney) and feces in the mice peritonitis‐sepsis infection model. *P* values were determined by Mann‐Whitney U test. (D) Statistical analysis of inflammatory cytokines after different treatments. Levels of IL‐6, TNF‐α, IL‐10 and IL‐4 in liver tissue. One‐way ANOVA was used to determine statistical significance (****p < 0.0001). (E) In intestinal infection model, bacterial suspension (6.0 × 10^8^ CFUs) was gavaged into the female ICR mice (n = 8 per group) and treated with colistin (0.5 mg/kg), ranitidine (50 mg/kg), or their combination at 1 h after infection. After 24 h post‐infection, bacterial loads in mouse liver, spleen and kidney and feces were determined. The experiments were conducted with six biological replicates and data were presented as mean ± SD. Statistical significance was analyzed by ordinary one‐way ANOVA (****p < 0.0001).

Next, we established two animal models of infection to investigate the in vivo effects of the combined action of ranitidine and colistin (Figure 8A). In a murine peritonitis‐sepsis model, monotherapy with colistin failed to rescue infected mice, whereas the combination therapy resulted in a 62.5% survival rate (Figure 8B). In line with these findings, the combination treatment group exhibited reduced bacterial burdens in the heart, liver, spleen, kidneys, and feces compared to those receiving colistin alone (Figure 8C). Additionally, we quantified inflammatory mediators across treatment groups using ELISA analysis. Strikingly, the combination therapy group exhibited significantly diminished levels of pro‐inflammatory cytokines, including tumor necrosis factor‐α (TNF‐α) and interleukin‐6 (IL‐6), and increased levels of anti‐inflammatory cytokines IL‐4 and IL‐10, relative to the monotherapy group (Figure 8D). To assess the in vivo propensity for resistance selection, isolates recovered from mice on days 3, 5 and 7 post‐treatment were analyzed by checkerboard assays. The ranitidine‐colistin combination retained comparable synergistic activity against all isolates (Figure S14), indicating that the regimen exerted no selective pressure for resistance emergence during the observation window.

Furthermore, we tested the efficacy of colistin and ranitidine in a mouse model of intestinal infection. The combination therapy significantly lowered bacterial loads in the liver, spleen, kidney, and feces by approximately 2‐log10 compared to colistin monotherapy (Figure 8E). Histopathological analysis revealed marked inflammatory cell infiltration in the livers of the control and monotherapy groups. The spleens from these groups exhibited disorganized white pulp with indistinct boundaries. In addition, renal lesions, including glomerular deformation and tubular hemorrhage, were observed. In contrast, no apparent pathological alterations were detected in the organs of mice receiving the combination treatment (Figure S15). Collectively, these data substantiate the in vivo efficacy of ranitidine in enhancing the activity of colistin against drug‐resistant bacterial infections.

## 3 Discussion

Colistin is a last‐resort antibiotic for treating MDR Gram‐negative bacterial infections. However, the dissemination of the plasmid‐borne transmissible colistin resistance genes *mcr‐1* and its variants [42] has compromised its clinical efficacy. Novel strategies such as the antibiotic adjuvant approach are urgently required to overcome colistin resistance. Current in vitro studies on colistin adjuvants are predominantly conducted under neutral conditions, yet intestinal pathogens such as *E. coli* and *Salmonella* must withstand acidic environments during host invasion [30]. These bacteria first encounter the stomach, a natural antibiotic barrier with a pH as low as 1.5‐2.5, and then the weakly acidic environment of the small intestine (pH 4.0‐6.0), where they face organic acids produced by the indigenous gut microbiota [43]. Consequently, bacteria that survive the gastric acid barrier can proliferate rapidly in the weakly acidic intestine.

The bacterial cell membrane plays a crucial role in acid stress resistance. Membrane lipids and proteins are involved in acid resistance mechanisms [44, 45, 46], primarily by inhibiting proton influx and maintaining membrane integrity [47]. Additionally, lipopolysaccharide (LPS) on the cell membrane is essential for bacterial adaptation to acidic conditions [48]. For instance, the inner membrane (IM)‐dependent two‐component systems, such as PmrAB, regulate the expression of membrane enzymes ArnT and EptA, which reduce the negative charge of LPS at low pH, thereby enhancing acid resistance [49]. Hence, there is a critical need to develop therapeutic strategies that can effectively target and eliminate *mcr*‐positive strains under acidic conditions.

In this study, we successfully identified a critical gene, *yqgB*, that could resensitize *mcr*‐positive bacteria to colistin treatment under acidic environment using TraDIS [50]. This integrative approach marries phenotype‐based selection with genomics, enabling the simultaneous assessment of all genes' roles in survival under selective pressures [51]. YqgB encodes a highly conserved small protein, which is implicated in acid stress response [52]. Previous studies showed that *yqgB*, together with *yqfZ*, can enable bacteria to cope with the physical environment of host and promote colonization [53]. Although its biochemical roles remain elusive, sequence and network analysis suggest that YqgB may interact with membrane homeostasis or membrane stress‐related proteins and is governed by stress‐related transcription factors [54]. Our mechanistic investigations revealed that, under acidic conditions, the deletion of *yqgB* in *mcr*‐positive bacteria downregulates fatty acid synthesis, altering membrane fluidity and enhancing colistin sensitivity. This is corroborated by related studies indicating that the upregulation of these genes bolsters bacterial acid resistance [55]. Furthermore, we demonstrated that *yqgB* absence in *mcr*‐positive bacteria led to an increase in the negative charge of LPS and the accumulation of ethanolamine, ultimately enhancing the bactericidal effect of colistin under weakly acidic environment.

Furthermore, we screened dual inhibitors of both MCR and YqgB proteins through deep learning approach. Traditional gene‐targeted drug design is arduous and time‐consuming. However, the advent of advanced computational techniques in virtual screening and drug discovery has paved new avenues. Current research is focused on drug discovery, molecular docking [56], molecular dynamics simulation [57], pharmacokinetic analysis, machine learning algorithms, and deep neural networks [58]. These cutting‐edge technologies can dissect the physicochemical interactions between molecules and targets, facilitating the discovery of novel bioactive compounds. For instance, Yao et al. identified a novel BfmR inhibitor through structural virtual screening, reviving carbapenem sensitivity in carbapenem‐resistant *Acinetobacter baumannii* [59]. Feng et al. employed artificial intelligence to uncover a new class of synthetic antimicrobials designed to counter bacterial resistance [60]. In this work, we virtually screened 7,590 small molecules to identify H_2_ receptor antagonists as potential dual inhibitors of MCR and YqgB, confirmed by molecular docking analysis to bind specifically to their active sites. Subsequent experiments confirmed the synergistic efficacy of H_2_ receptor antagonists with colistin against *mcr*‐positive bacteria across neutral and acidic conditions.

Colistin's significant nephrotoxicity and neurotoxicity in mammals constrain its clinical use [61]. Thus, the deployment of low‐dose colistin and the development of colistin detoxifiers are imperative for clinical application. For instance, minocycline has been shown to mitigate colistin‐induced neurotoxicity by curbing oxidative stress and mitochondrial dysfunction [62]. Importantly, our study reveals that ranitidine, as a colistin enhancer, can safely reduce the colistin dosage to 0.5 mg kg^−1^, significantly below the clinical recommendation. Moreover, our research underscores the ability of ranitidine and famotidine to curb the evolution and dissemination of drug resistance.

In conclusion, our study identifies the acid stress response gene *yqgB* mediating the improved susceptibility of *mcr*‐positive bacteria to colistin, unveiling an unexpected interplay between acid resistance system and colistin resistance. Leveraging deep learning‐driven drug screening, we identified H_2_ receptor antagonists as dual inhibitors of MCR and YqgB, synergizing with colistin against *mcr*‐harboring bacteria in both neutral and acidic conditions. In animal models of infection, the combination therapy of ranitidine and colistin demonstrates remarkable efficacy against systemic and intestinal infections caused by *mcr*‐positive Gram‐negative pathogens.

## 4 Materials and Methods

### 4.1 Bacterial Strains and Reagents

In Table S3, all strains used in this study are mentioned. Bacteria were stored in nutrient broth containing 20% (v/v) glycerol at ‐80°C. All strains were cultured on Mueller‐Hinton broth (MHB) or LB agar (LBA) plates for the following tests. Chemical reagents were procured from Aladdin in Shanghai, while antibiotics were purchased from the China Institute of Veterinary Drug Control.

### 4.2 Generation of the Transposon Mutant Library

Tn5 insertion mutants were generated in *S*. 4,[5],12:i‐ (*mcr‐3*) using the pUTmini‐Tn5km2 plasmid (Amp^R^ Km^R^). Briefly, pUTmini‐Tn5km2 plasmids were purified from clones and individually transformed into the donor strain *E. coli* X7213. Conjugation was performed using *S*. 4,[5],12:i‐ (*mcr‐3*) as the recipient bacterium and defective strain *E. coli* X7213 carrying pUTmini‐Tn5km2 plasmid as the donor bacterium. The donor and recipient bacteria were cultured to an OD_600_ of 1.0, then the bacteria were collected by centrifugation and cleaned 3 times with phosphate‐buffered saline (PBS). The recipient and donor bacteria were suspended in 500 µL LB broth, mixed evenly, and then incubated for 6 h at 37°C onto the center of LB agar plate containing 2,6‐diaminopimeilc acid (DAP). After incubation, the bacteria on the plate were washed with 2 mL PBS and evenly coated on LBA plates containing kanamycin. After overnight incubation at 37°C, the total bacterial count was estimated by calculating the proportion of each plate in multiple plates. Each batch contained approximately 10 000 mutants, and by collecting 20 mutant batches, a total of 200 000 transconjugants are generated. This transposon mutation library was used as input library.

### 4.3 Colistin Selections on the Library

The above‐mentioned transposon mutation library was treated with 1 × MIC colistin at 37°C for 12 h, and the bacteria were collected by centrifugation. After resuspending in PBS, the samples were coated onto LBA plates containing both colistin and kanamycin and incubated overnight at 37°C. The next day, the bacteria were collected as output. DNA was then extracted by using a TIANamp bacterial DNA kit (TianGen, Beijing, China) following the manufacturer's instruction. TraDIS‐specific sequencing was performed on a HiSeq 2500 machine using 50‐bp single‐end reads to produce transposon‐directed reads as described previously [51].

### 4.4 TraDIS Sequencing and Bioinformatics Analysis

To determine the insertion sites of each transposon that had randomly inserted into the genome of each mutant, a TraDIS library was prepared using specifically designed TraDIS adapters for the Tn5 transposon. This method increased the enrichment of genuine transposon‐chromosome junctions by preventing hybridization of the reverse primer [63]. This ensured that the first 10 bp of every read consisted of transposon sequence and that the remaining sequence was downstream of where the transposon was inserted. These reads were then mapped using SMALT (WTSI). The change in the number of reads that mapped to each gene between the control and the selections was measured using Log_2_FC (fold change). Log_2_FC was used as a measure of comparison of fold changes in read numbers compared to the Input control; ≥ 2 and ≤ ‐2 were used as cutoff values for genes with different numbers of insertions.

### 4.5 Mutant Construction

Mutants were constructed using the suicide vector pRE112. All oligonucleotide primers used in this study were purchased from GENEWIZ (Nanjing, China). All mutants were created using the *Salmonella enterica* serovar Typhimurium (STm) 14028s (pUC19‐*mcr‐3*). The constructed strains and mutations were confirmed by PCR and DNA sequencing.

### 4.6 MIC Determinations

Minimum inhibitory concentrations (MICs) of drugs were measured using the standard broth microdilution method with reference to CLSI 2021. Briefly, overnight culture of bacteria was diluted to MHB in a 1:1000 ratio. Next, equal amounts of medicines at varying concentrations were combined with the bacterial suspension (1.5 × 10^6^ CFUs/mL) in a sterile 96‐well microtiter plate. MIC values were determined as the lowest medication concentration at which no detectable bacterial growth occurred after 18 h of incubation at 37°C.

### 4.7 Time‐dependent Killing Curves

Overnight cultures of bacteria were diluted 1/1000 in MHB. Then, the bacteria were treated with different concentrations of drugs in different pH environments. At each time point (0, 4, and 8 h), samples were serially diluted ten‐fold and plated on MHA plates. After bacterial colonies were incubated for a whole night, the number of viable CFUs/mL was calculated [64].

### 4.8 Confocal Laser Scanning Microscopy

Bacterial suspensions (10^6^ CFUs/mL) were cultured for 1 h at 37°C with different concentrations of drugs in different pH environments. Then, PI (5 mM, 3 µL) and SYTO 9 (0.835 mM, 4 µL) were added to bacteria and incubated in dark for 15 min. The glass slide was filled with 20 µL of bacterial suspension, fixed with 4% glutaraldehyde, and examined under a CLSM microscope (Leica TCS SP2, Heidelberg, Germany).

### 4.9 Flow Cytometry Assay

The bacterial suspension was detected by flow cytometry after staining with PI and SYTO 9 as mentioned above. About 100,000 ungated events were measured by the CytExpert Flow Cytometer (Beckman, USA), and the data were processed using CytExpert 2.0 software [65].

### 4.10 RT‐qPCR Analysis

The knockout and original bacteria were incubated at pH = 5 for 4 h. After incubation, total RNA was extracted and measured using a 260/280 nm absorbance ratio. cDNA synthesis was performed by reverse transcription of 1 µg extracted RNA using the HiScript III RT SuperMix for qPCR (+gDNA wiper) (Vazyme, Nanjing, China). Using the ChamQ Universal SYBR qPCR Master Mix (Vazyme, Nanjing, China), RT‐qPCR analysis was carried out in a LineGene 9600 Plus Real‐Time PCR System (Bioer, Hangzhou, China). Using a relative quantitative method, the fold changes in mRNA expression with respect to the reference genes (16S rRNA) in bacteria were calculated.

### 4.11 Detection of Arginine Decarboxylase (ADC), Agmatine Ureahydrolase (AGMAT), Ethanolamine, Phosphatidylethanolamines (PE) and Spermidine Content

Bacteria cultured overnight were diluted in a 1/100 ratio and grown for 4 h. The bacteria were collected by centrifugation and were resuspended with PBS to adjust an OD_600_ of 0.5. Then, colistin with different concentrations was added under different pH environments for 4 h. Arginine decarboxylase, agmatine ureohydrolase, and spermidine content were detected using corresponding enzyme‐linked immunosorbent assay (ELISA) kits. Briefly, a 96‐well plate was filled with 50 µL of the sample or standard sample, and then the antibody enzyme complex was added. Following five rounds of washing, 50 µL of chromogenic solution was added and incubated for 10 min at 37°C, then the termination solution was added. At 450 nm, the absorbance was measured.

### 4.12 ΔpH Measurement

Overnight STm 14028s (pUC19‐*mcr‐3*) and STm 14028s Δ*yqgB* (pUC19‐*mcr‐3*) were resuspended to an OD_600_ of 0.5 with PBS. Then, pH‐sensitive fluorescent probe BCECF‐AM with final concentration of 2 × 10^−6^ m was added to the bacterial culture [66]. Next, the bacterial mixture was treated with different concentrations of colistin (0, 0.5, 1 µg/mL) at different pH environments (pH = 5, pH = 3) for 1 h at 37°C. The fluorescence intensity was measured within 20 min with an excitation wavelength of 488 nm and an emission wavelength of 535 nm.

### 4.13 Bacterial PMF Detection

Briefly, 1 mL of STm 14028s (pUC19‐*mcr‐3*) and STm 14028s Δ*yqgB* (pUC19‐*mcr‐3*) (OD_600_ = 0.5) was collected and diluted to 10^6^ CFUs/mL. Then, the bacterial suspension was treated with CCCP, COL (0.5 µg/mL) for 1 h at 37°C. Then, 10 µL of 3 mM DiOC_2_(3) (3,3'‐diethyl‐oxo‐iodocarbocyanine) was added to the bacterial suspension and incubated at 37°C for 30 min. CytExpert Flow Cytometer (Beckman, USA) was used to measure signal strength and FlowJo V10.8.1 was used for analysis. Green fluorescence was detected using a bandpass filter with a bandwidth of 488 to 530 nm, and red fluorescence was detected using a bandpass filter with a bandwidth of 488 to 610 nm. The PMF was calculated and normalized as the ratio of the red/green fluorescence intensity. Membrane potential is calculated by the following formula: PMF = Lg (10^3/2^ × (red fluorescence / green fluorescence)).

### 4.14 Transcriptomic Analysis

The knockout (STm 14028s Δ*yqgB* (pUC19‐*mcr‐3*)) and original bacteria (STm 14028s (pUC19‐*mcr‐3*)) were incubated for 4 h under weakly acidic environment (pH = 5). Following incubation, the samples' total RNA was extracted, measured with a Nanodrop spectrophotometer from Thermo Scientific (MA, USA) and sequenced using an Illumina Hiseq 2000 system from Majorbio (Shanghai, China) [67].

### 4.15 Determination of Membrane Charge

FITC‐labeled Poly‐L‐Lysine (PLL) was used to evaluate the membrane charge of both knockout and original bacteria [68]. First, the bacteria were treated with different concentrations of colistin (0, 0.5, 1 µg/mL) and acidic environment (pH = 5, pH = 3) at 37°C for 4 h, followed by washing with PBS and resuspending. Then, the bacterial suspension (300 µL) was incubated at room temperature for 10 min in tris buffer (0.03 M, pH = 8.0), 20% sucrose, and 20 µg/mL FITC‐PLL solution. The bacteria were then subjected to three cycles of centrifugation followed by resuspension in tris solution with 20% sucrose in order to eliminate any unbound PLL. Subsequently, 200 µL of spheroplast sample was placed into the wells of a black‐walled microtiter plate, and a microplate reader was used to assess the FITC fluorescence, utilizing an excitation wavelength of 490 nm and an emission wavelength of 525 nm. A rise in the fluorescence reading signifies a reduction in the positive charge present on the cell membrane's surface.

### 4.16 Bacterial Count Detection in the Presence of Ethanolamine

The STm 14028s (pUC19‐*mcr‐3*) count was adjusted to approximately 10^6^ CFUs/mL and then divided into new EP tubes. Afterward, 1 × MIC of colistin and different concentrations of ethanolamine (0, 5, 10, 20 µg/mL) were added to the bacteria and adjusted to different pH environments (pH = 5, pH = 3). After incubating for 3 h, samples were serially diluted ten‐fold and plated on LBA plates. After bacterial colonies were incubated for a whole night, the number of CFUs/mL were calculated.

### 4.17 Cell Membrane Permeability

The fluorescent dyes propidium iodide (PI) were used to evaluate the permeability of cell membrane. [69]. Briefly, the bacterial suspension of STm 14028s (pUC19‐*mcr‐3*) and STm 14028s Δ*yqgB* (pUC19‐*mcr‐3*) with an OD_600_ = 0.5 was incubated with PI (5 µM) at 37°C for 30 min. Subsequently, bacterial mixture treated with different concentrations of colistin (0, 0.5, 1 µg/mL) was added in different pH environments (pH = 5, pH = 3). Using the Microplate reader (Tecan) with an excitation wavelength of 535 nm and an emission wavelength of 615 nm, the fluorescence values were measured after 1 h incubation period.

### 4.18 β‐Galactosidase Activity Assay

The bacteria STm 14028s (pUC19‐*mcr‐3*) and STm 14028s Δ*yqgB* (pUC19‐*mcr‐3*) cultured overnight were expanded at a ratio of 1/100 for 4 h and then collected by centrifugation. The bacterial cultures were resuspended with PBS and adjusted for OD_600_ = 0.5 and sub‐packed into new EP tubes. The bacterial suspension was treated with different concentrations of colistin (0, 0.5, 1 µg/mL) for 4 h under different pH conditions (pH = 5, pH = 3). Afterward, bacteria were washed with PBS and centrifuged, 200 µL of supernatant was placed on 96‐well microtitration plates, and O‐nitrophenyl‐D‐galactopyranoside (ONPG) at a final concentration of 3 mM was added and incubated at 37°C for 1 h. The absorbance at 420 nm was measured using an Infinite E Plex Microplate reader (Tecan).

### 4.19 Measurement of ROS Levels

The level of ROS in STm 14028s (pUC19‐*mcr‐3*) and STm 14028s Δ*yqgB* (pUC19‐*mcr‐3*) was measured using the fluorescent probe 2′,7′‐dichlorodihydro‐fluorescein diacetate (DCFH‐DA, 10 µM) [70] (Beyotime, Shanghai, China). To sum up, the bacterial culture was treated with the fluorescent probe DCFH‐DA and allowed to incubate for 30 min at 37°C. Afterward, the culture was washed twice with PBS. The bacterial mixture was cultured for1 h at 37°C after being treated with different concentrations of colistin (0, 0.5, 1 µg/mL) for 4 h under different pH conditions (pH = 5, pH = 3). The fluorescence values were measured by the Microplate reader (Tecan) with excitation wavelength of 488 nm and emission wavelength at 525 nm after 1 h incubation period.

### 4.20 Deep Learning‐Based Screening

The known MCR inhibitors were randomly partitioned into a training set and an independent test set at a ratio of 8:2, stratified by activity classes to ensure data distribution consistency. A quantitative structure‐activity relationship (QSAR) model was constructed using a graph neural network architecture. The network comprised a graph convolutional layer (64 nodes) with batch normalization and graph pooling operations, followed by a fully connected hidden layer (128 nodes) with additional batch normalization. To mitigate overfitting, a dropout rate of 0.25 was applied during training, alongside Adam optimization with a learning rate of 0.001. Model performance was rigorously evaluated through 5‐fold cross‐validation on the training cohort, with subsequent external validation using the held‐out test set.

Upon successful validation (AUC > 0.85, accuracy > 80%), the optimized QSAR model was used to screen 7,590 small molecules. 7,590 small molecules from the FDA‐approved compound library. After 7590 small molecules were input into the model, the model evaluated whether these small molecules have MCR inhibitory activity. Subsequently, small molecules with MCR inhibitory activity were output as 1, while those without MCR inhibitory activity were output as 0. Next, the small molecules with MCR inhibitory activity output in the model were further dock with MCR molecules. Small molecules with strong affinity (< ‐7.0 kcal/mol) after docking were predicted to exhibit ADMET properties, mainly including LogP, blood‐brain barrier penetration, LC50, etc. By predicting these characteristics, we selected small molecules with high safety. Subsequently, the selected small molecules were subjected to molecular docking with the YqgB protein. Those exhibiting high binding affinity to YqgB (< ‐7.0 kcal/mol) were chosen for experimental validation.

### 4.21 Checkerboard Assays

The Fractional Inhibitory Concentration Index (FICI) was assessed using checkerboard assays. Initially, 100 µL of Mueller Hinton broth (MHB) was added to each well of a 96‐well microtiter plate arranged in an 8 × 8 matrix. In the first row, 100 µL of colistin was added and subsequently diluted to the seventh row. The test compound was then added to the first column and diluted in the same manner to the seventh column. Following this, 100 µL of bacterial suspension, at a concentration of 10^6^ CFUs per well, was incorporated into each well. After incubating for 18 h, the absorbance of the resulting mixtures was read at 600 nm using a microplate reader. The Fractional Inhibitory Concentration Index (FICI) was calculated using the following formula: FIC index = FICIa + FICIb = MICab/MICa + MICba/MICb. Synergy is indicated by the FIC index of ≤ 0.5 [71].

### 4.22 Surface Plasmon Resonance (SPR) Assays

Surface plasmon resonance (SPR) was employed to assess the binding affinity between ranitidine and the MCR/YqgB protein. The purified recombinant MCR/YqgB protein was covalently attached to a CM5 sensor chip through standard amine‐coupling chemistry. Serial dilutions of ranitidine (0.3125‐10 µM) were freshly prepared in running buffer (PBS, pH 7.4, supplemented with 0.05% Tween‐20) and injected across the immobilized protein at 30 µL/min at 25°C. Association and dissociation kinetics were continuously monitored, and the chip surface was regenerated after each injection using 10 mM glycine‐HCl (pH 2.5). Binding parameters, including the equilibrium dissociation constant (KD), were determined by fitting the data to a 1:1 Langmuir interaction model.

### 4.23 Heteroresistance Frequency (HRF) Assays

The heteroresistance frequency (HRF) of *Klebsiella pneumoniae* YZ6 to colistin was evaluated by quantifying the proportion of bacterial subpopulations capable of growth under elevated colistin concentrations. Briefly, bacterial cultures were exposed to colistin at 2 ×, 4 ×, and 8 × MIC, either alone or in combination with ranitidine or famotidine. For each treatment condition, aliquots of appropriately diluted cultures were spread onto selective agar plates containing the indicated colistin concentrations, while parallel dilutions were plated onto non‐selective medium to determine total viable counts. After incubation, colony‐forming units (CFUs) were enumerated on both selective and non‐selective plates. The heterogeneous resistance frequency was calculated as the ratio of CFUs recovered on colistin‐containing plates to the total CFUs on drug‐free plates.

### 4.24 Biofilm Formation Determination

The impact of gene knockout on biofilm formation under acidic environment [72]. Knockout bacteria (STm 14028s Δ*yqgB* (pUC19‐*mcr‐3*)) and original bacteria (STm 14028s (pUC19‐*mcr‐3*)) were cultured under different acidic environment and treated with different concentrations of colistin (0 to 2 µg/mL).

The effect of adding famotidine and ranitidine on biofilm formation in neutral and acidic environment was accssed. *S*. 4,[5],12:i‐ (*mcr‐3*) was treated with colistin (0 to 2 µg/mL) alone or in combination with ranitidine or famotidine (5 mg/mL), and different environment were adjusted (pH = 7, pH = 5).

The bacteria were washed three times with 300 µL of PBS after 36 h of incubation at 37°C. Next, 200 µL of methanol was added and fixed for 15 min, then the fixative was sucked out and dried naturally. 0.1% crystal violet was added for 15 min. Then, the dye was extracted and washed three times with PBS, and air dried naturally. Finally, 100 µL of 33% acetic acid was added and the crystal violet was dissolved at 37°C for 30 min. Finally, the absorbance at 570 nm was measured by Infinite E Plex Microplate reader (Tecan).

### 4.25 Biofilm Eradication Assay

Overnight cultured *S*. 4,[5],12:i‐ (*mcr‐3*) was diluted 1/100 in MHB and the mixture was cultured at 37°C for 4 h. Then, in a 96‐well microtitration plate, 100 µL of bacterial suspension was mixed with an equal volume of MHB and cultured in the 37°C environment for 36 h to form biofilm. Then, the bacterial suspension was discarded and each well was treated with colistin alone or in combination with ranitidine/famotidine for 2 h. After incubation, ultrasound treatment was used for 20 min. The mixture was then diluted in PBS and incubated on LB agar plates at 37°C. After incubation for 18 h, colonies were counted accordingly.

### 4.26 Mutation Preventive Concentration (MPC) Assay

LB agar plates were prepared with varying pH levels (pH = 7 and pH = 5), and treated with colistin alone or in combination with ranitidine or famotidine at concentrations ranging from 0 to 4 mg/mL. Subsequently, 100 µL of bacterial suspension at a concentration of 1.0 × 10^10^ CFUs was applied to the LB agar plates, which were then incubated at 37°C for 72 h. After incubation, the Minimal Preventive Concentration (MPC) was assessed by examining bacterial growth on the plates. The MPC represents the lowest concentration capable of inhibiting the development of resistance (mutant colonies) [73].

### 4.27 Conjugation Assays

Conjugation experiments were carried out by observing the conjugative transfer frequency between the donor and recipient bacteria, under conditions with or without ranitidine or famotidine [74]. The *mcr‐1* donors utilized were *E. coli* LD67‐1 and *E. coli* CSZ4, with *E. coli* EC600 containing the rifampicin resistance gene as the recipient strain. The above bacteria cultured overnight were diluted at a ratio of 1:100 and cultured in LB broth for 4 h. The OD_600_ of the donor and recipient bacteria was then adjusted to 0.5. A total volume of 2 mL, with a 1:1 ratio of donor to recipient, was prepared, to which varying concentrations of ranitidine or famotidine (ranging from 0 to 8 mg/mL) were introduced. Following an incubation period of 18 h at 37°C, the mixture underwent serial dilution and was plated on LB agar containing single or double antibiotics. The determination of conjugators and the assessment of conjugation frequencies were conducted through bacterial CFUs counting.

### 4.28 Swimming Motility Experiment

Agar media at a concentration of 0.3% (w/v), consisting of trypticase peptone (10 g/L), sodium chloride (10 g/L), and yeast extract (5 g/L), were employed to evaluate the swimming motility of bacteria [69]. Agar media was prepared at different pH and supplemented with different colistin (0 to 2 µg/mL) alone or in combination with ranitidine or famotidine (5 mg/mL). Then, 2 µL of *S*. 4,[5],12:i‐ (*mcr‐3*) with an OD_600_ of 0.5 was injected into the center of the plate. The agar plates were then placed in 37°C culture for 48 h. Finally, the swimming ability of bacteria was evaluated by calculating the area of the microsphere.

### 4.29 Safety Assessment

The hemolysis of colistin alone and in combination with the compound was evaluated [75]. In summary, 8% of blood cells were incubated at 37°C for 1 h with varying concentrations of colistin ranging from 0 to 8 µg/mL, either individually or mixed with 5 mg/mL of the compound. To serve as positive and negative controls, Triton X‐100 (0.2%) and phosphate‐buffered saline (PBS) were used, respectively. Following the incubation period, the samples were centrifuged, and the absorbance of the supernatant at 576 nm was recorded using an Infinite E Plex Microplate reader (Tecan). Finally, the hemolysis rate (%) was calculated accordingly.

Acute toxicity in mice: colistin (0.5 mg/kg), colistin + ranitidine (0.5 mg/kg + 50 mg/kg) was intra‐peritoneally injected into mice (n = 6 per group). Mice were inspected for one week. Blood from mice was collected on the seventh day for whole‐blood cell analysis.

### 4.30 Ethical Statement

This study was performed according to the relevant guidelines of Jiangsu Laboratory Animal Welfare and Ethical of Jiangsu Administrative Committee of Laboratory Animals (SYXK‐2022‐0044). All animal experiments were approved by the Animal Care Committee of Yangzhou University.

### 4.31 Mouse Peritonitis‐Sepsis Infection Model

*S*. 4,[5],12:i‐ (*mcr‐3*) suspension (6.0 × 10^8^ CFUs) was intraperitoneally injected into female ICR mice (n = 8 per group). 1 h after infection, mice were intraperitoneally injected with colistin (0.5 mg/kg), ranitidine (50 mg/kg), or a combination of both (0.5 + 50 mg/kg) for treatment. This experiment was conducted twice, once to monitor the survival rate of seven days mice. Another experiment was conducted to dissect and calculate the bacterial load of each organ 24 h after treatment.

### 4.32 Mouse Intestinal Infection Model

Female ICR mice (n = 8 per group) were gavaged with 6.0 × 10^8^ CFUs *S*. 4,[5],12:i‐ (*mcr‐3*). Colistin (0.5 mg/kg), ranitidine (50 mg/kg), or a combination of both (0.5 + 50 mg/kg) was injected intraperitoneally into the mice 1 h following the infection. The bacterial counts in the organs and feces of the mice were measured 24 h after infection.

### 4.33 Measurement of Inflammatory Factors and Histological Analysis

Dissected liver tissue was rinsed in sterile PBS. According to the manufacturer's instructions, the levels of TNF‐α, IL‐6, IL‐10 and IL‐4 were measured using the corresponding ELISA kits (Enzyme‐linked Biotechnology, Shanghai). The dissected organs (liver, spleen, kidney) were fixed with 4% paraformaldehyde and the pathological changes of the tissues were observed by H&E staining.

### 4.34 Statistics and Reproducibility

Statistical analyses were performed using GraphPad Prism 9.0. Data are presented as mean ± SD. Unpaired *t*‐test or standard one‐way or two‐way ANOVA for multiple comparisons were carried out following the in vitro testing. In the mouse models, the significance of survival rates and bacterial loads were analyzed by the log‐rank (Mantel‐Cox) test or the Mann‐Whitney U test, respectively. The calculated *p* < 0.05 was defined as a significant difference (*p < 0.05, **p < 0.01, ***p < 0.001, and ****p < 0.0001).

## Author Contributions

Y.L. conceived, designed, and supervised the study. J.C., M.H., J.L., S.Z., T.Z. and J.L. conducted experiments. Y.L., Z.W. and J.C. analyzed the data and wrote the manuscript. All authors read and approved the final version of the paper.

## Conflicts of Interest

The authors declare no conflicts of interest.

## Supporting information




**Supporting File**: advs74558‐sup‐0001‐SuppMat.docx.

## Data Availability

The raw sequence data generated during this study have been deposited in the NCBI Sequence Read Archive (SRA) under the Bioproject PRJNA1424243. All other relevant data supporting the findings of this study are available from the corresponding author upon reasonable request.
