# Supplementary material for: Discovery of H2 Receptor Antagonists as Colistin Enhancers by Targeting Acid Stress Response
Source: Adv Sci (Weinh). 2026 Feb 25;13(26):e14165. doi: 10.1002/advs.202514165 (PMC13159126; doi:10.1002/advs.202514165)
Supplement: Supplementary file 1 — Supporting File: advs74558‐sup‐0001‐SuppMat.docx. [file ADVS-13-e14165-s001.docx]

**Supplementary Materials**

**Supplementary Figures**


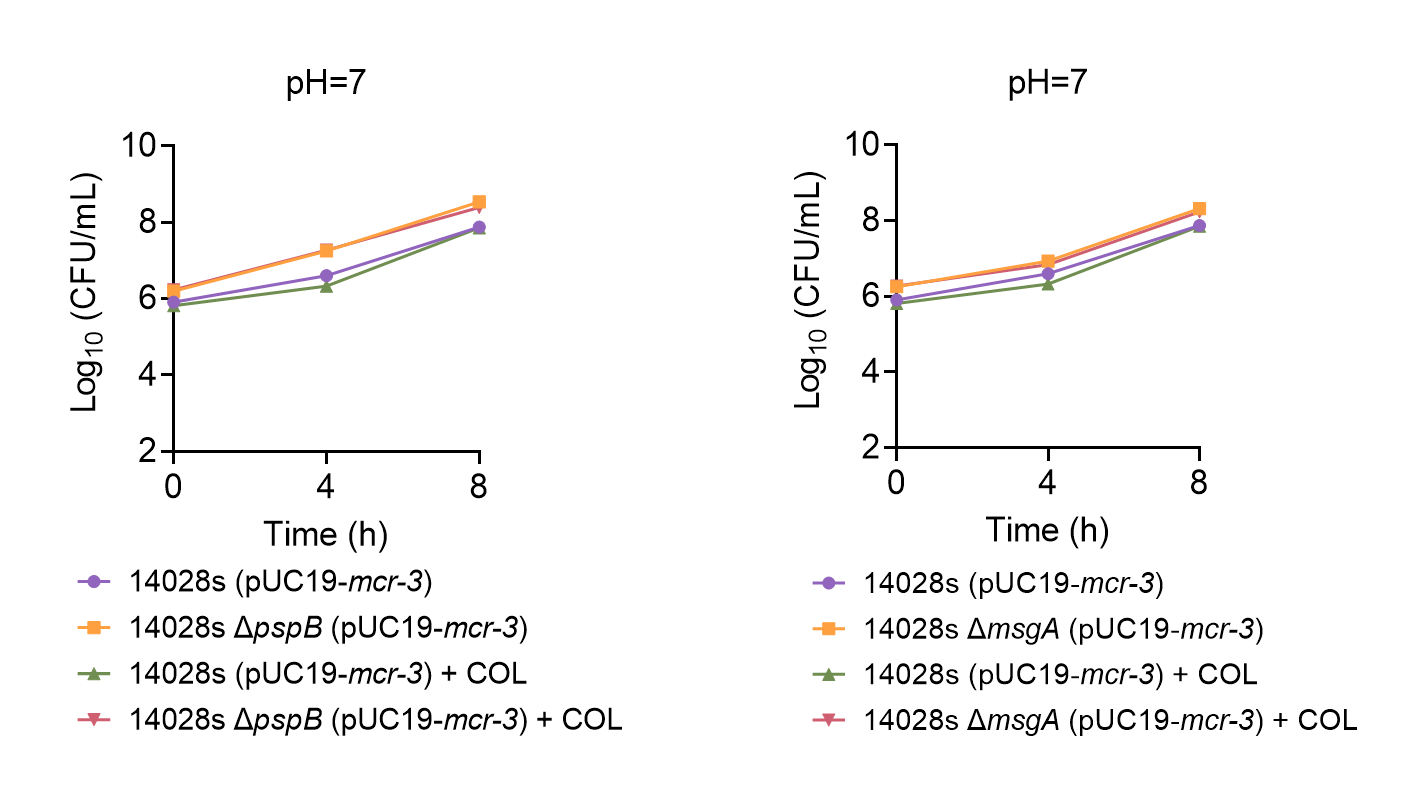


**Figure S1. Time-dependent killing curves of *pspB* and *msgA*-deficient strains upon colistin treatment under neutral environment**.

Data were presented as mean ± SD from three independent experiments.

**
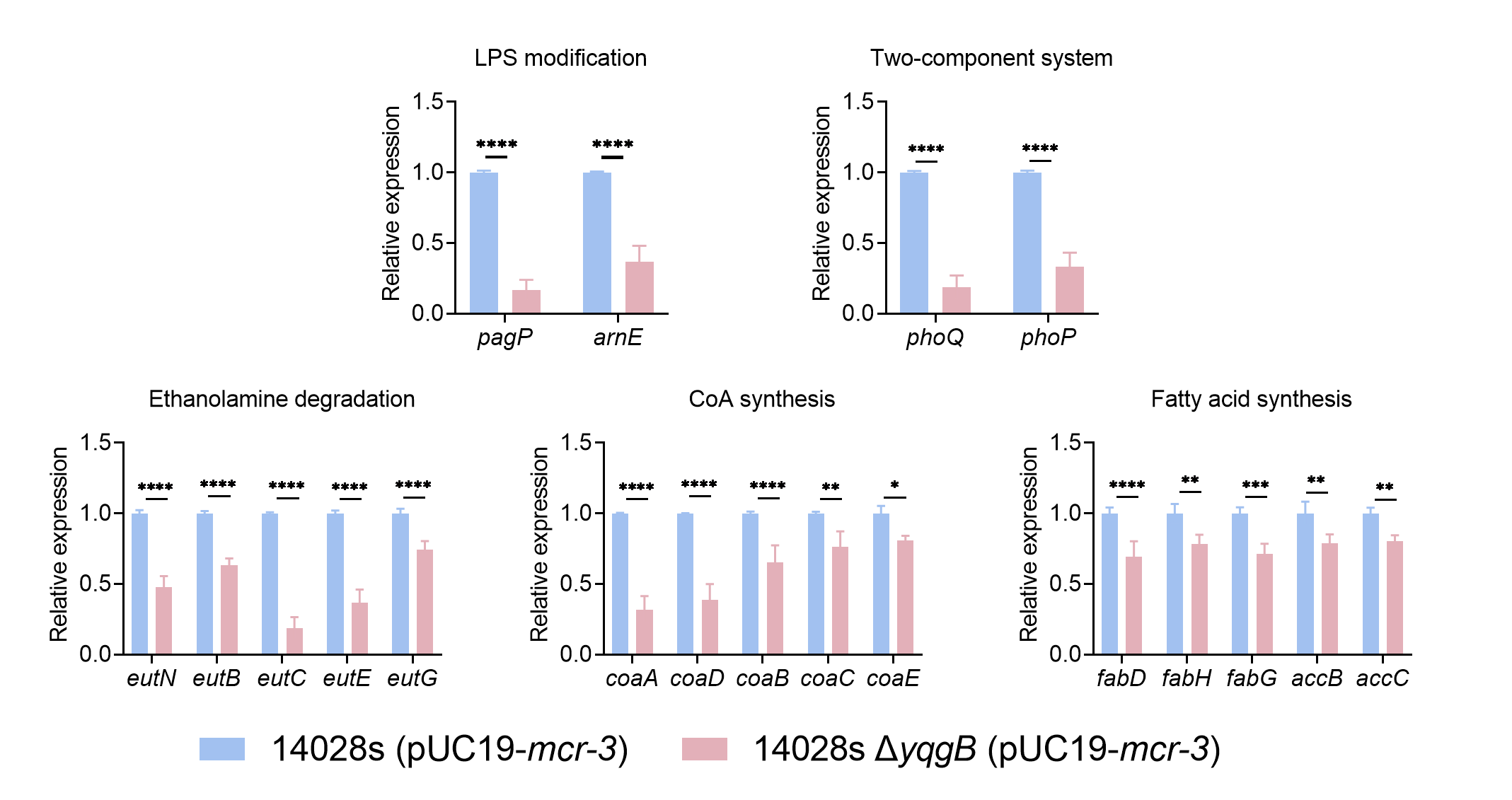
**

**Figure S2. The effect of *yqgB* deletion on the expression of representative genes in *mcr*-positive bacteria under weakly acidic environments.**

RT-qPCR analysis of the expression of representative DEGs from transcriptome results. Data were presented as mean ± SD from three independent experiments (**P* < 0.05, ***P* < 0.01, ****P* < 0.001, *****P* < 0.0001).


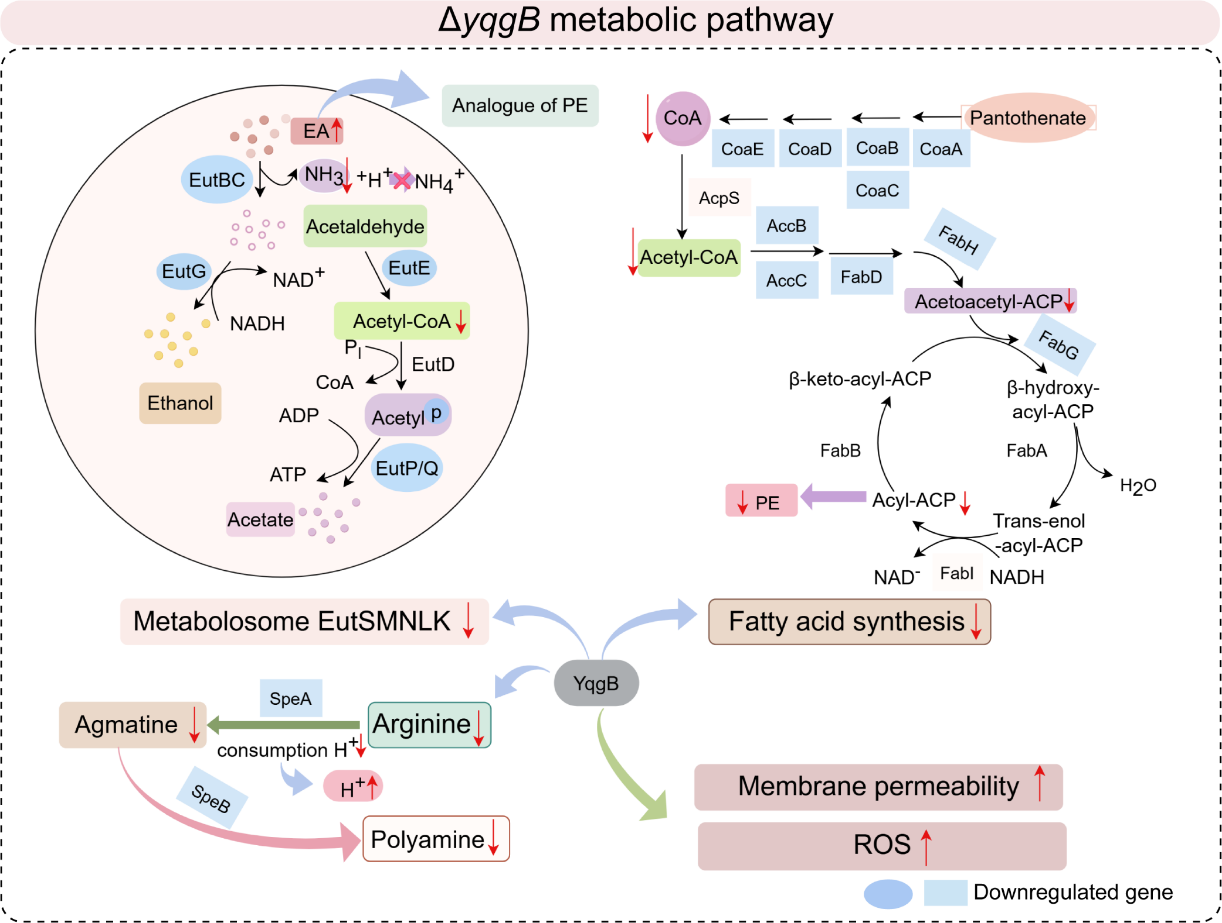


**Figure S3. Schematic diagram of gene regulation pathways in *yqgB*-deficient strains.** Genes in the blue box indicate downregulation.


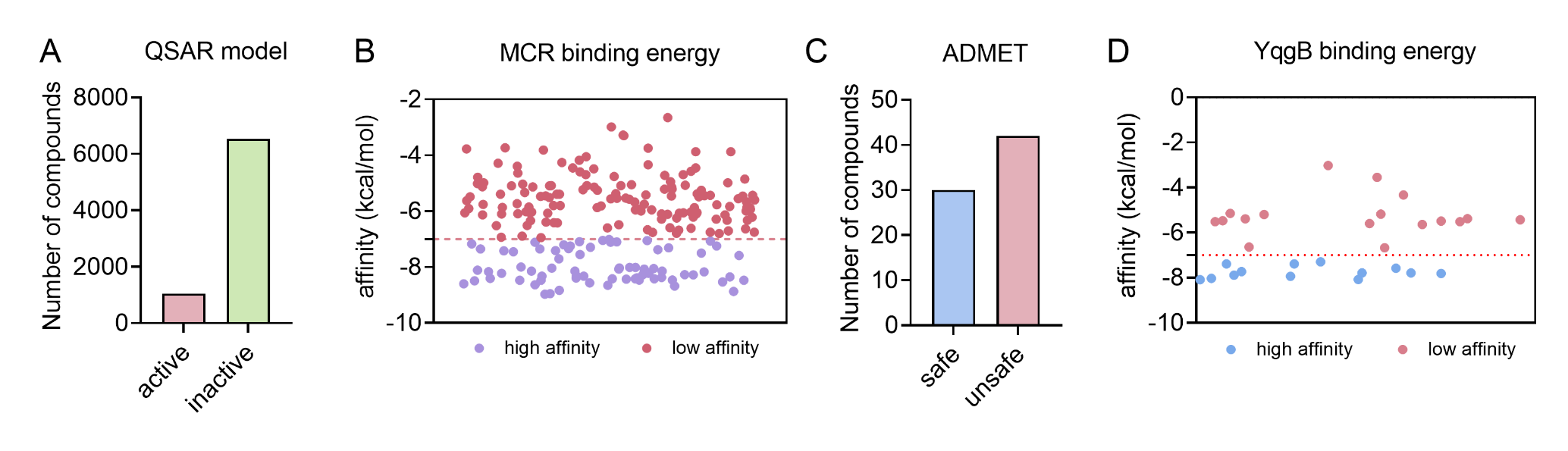


**Figure S4. Virtual screening of small molecules capable of inhibiting MCR and YqgB proteins**

(A) 7590 small molecules were predicted by the QSAR model to inhibit the activity of MCR protein. (B) Binding energy analysis of molecular docking between small molecules and MCR protein. (C) Safety analysis of small molecules by ADMET. (D) Binding energy analysis of molecular docking between small molecules and YqgB protein. Molecules with binding energy below -7.0 kcal/mol are considered to have strong affinity.


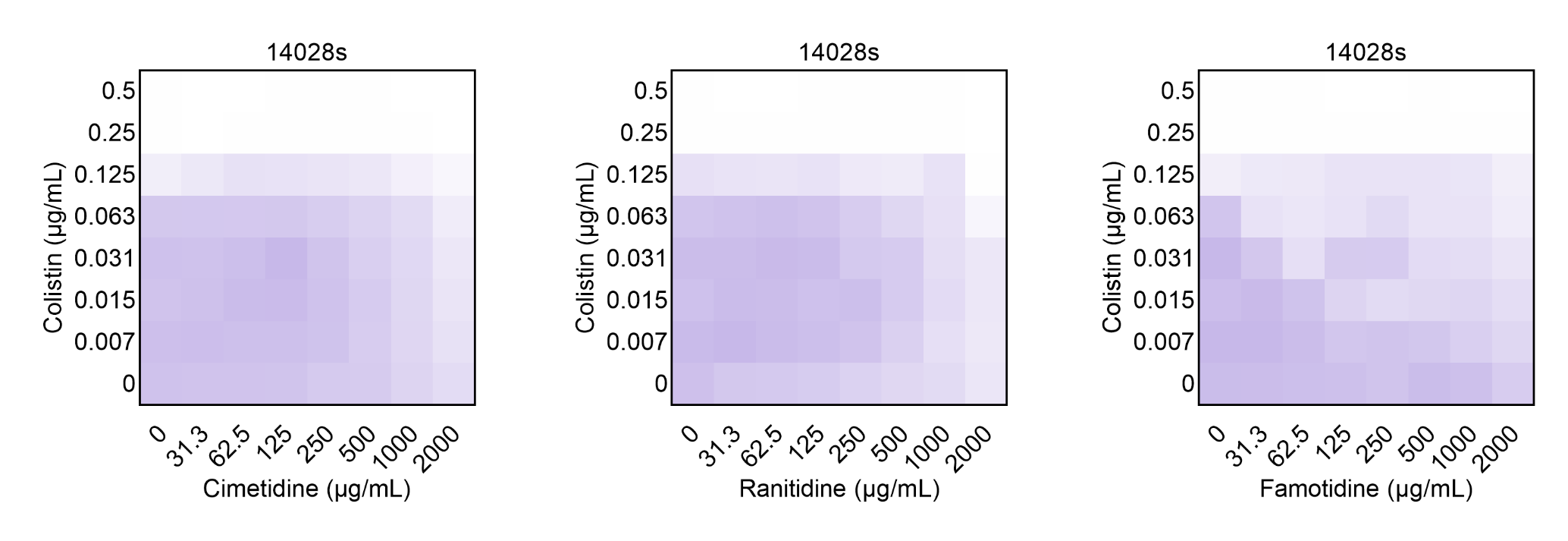


**Figure S5. Checkerboard broth microdilution assays between cimetidine/ranitidine/famotidine and colistin against *mcr*-negative bacteria**.

Dark purple regions represent higher bacterial cell density. The mean OD at 600 nm of two biological replicates was shown.


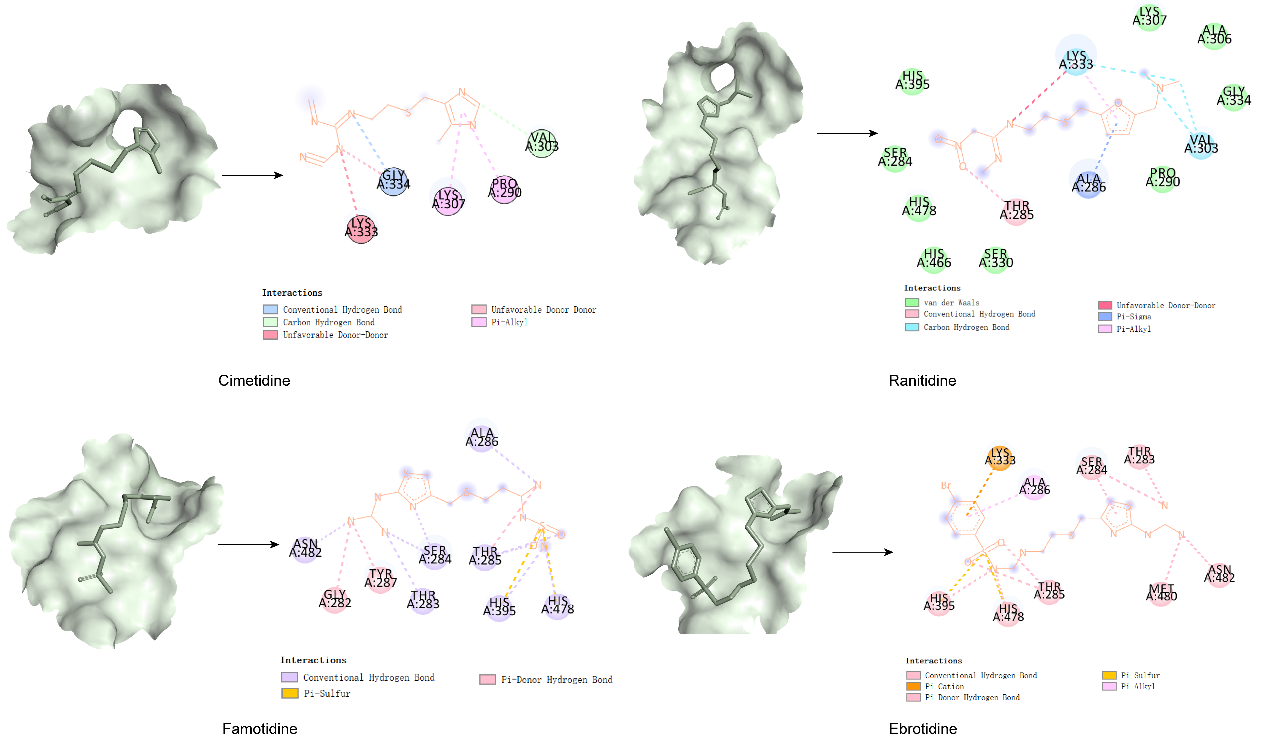


**Figure S6. Molecular docking analysis of cimetidine/ranitidine/famotidine/ ebrotidine and MCR protein.**

The interactions and binding sites in MCR were shown using a two-dimensional diagram.


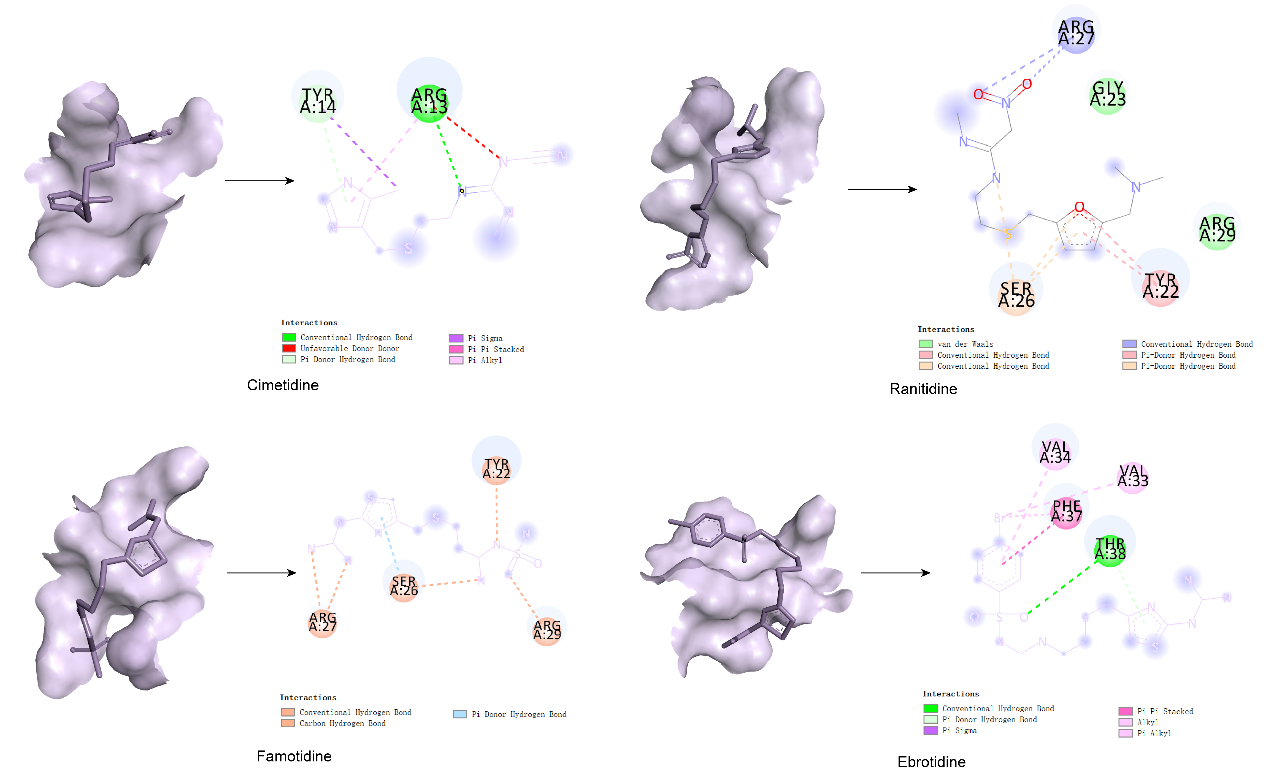


**Figure S7. Molecular docking analysis of cimetidine/ranitidine/famotidine/ ebrotidine and YqgB protein.**

The interactions and binding sites in YqgB were shown using a two-dimensional diagram.


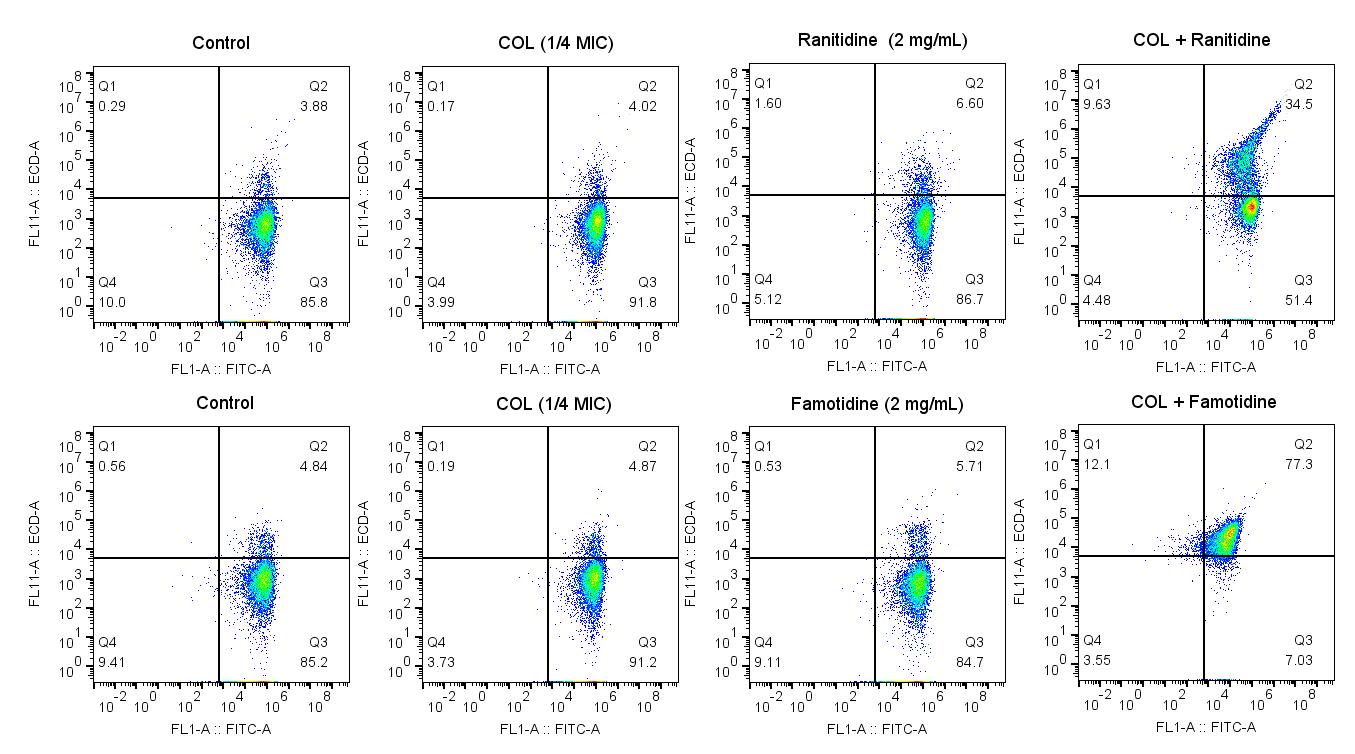


**Figure S8. Flow cytometry analysis of the proportion of live and dead bacteria treated with monotreatment or combination treatments.**


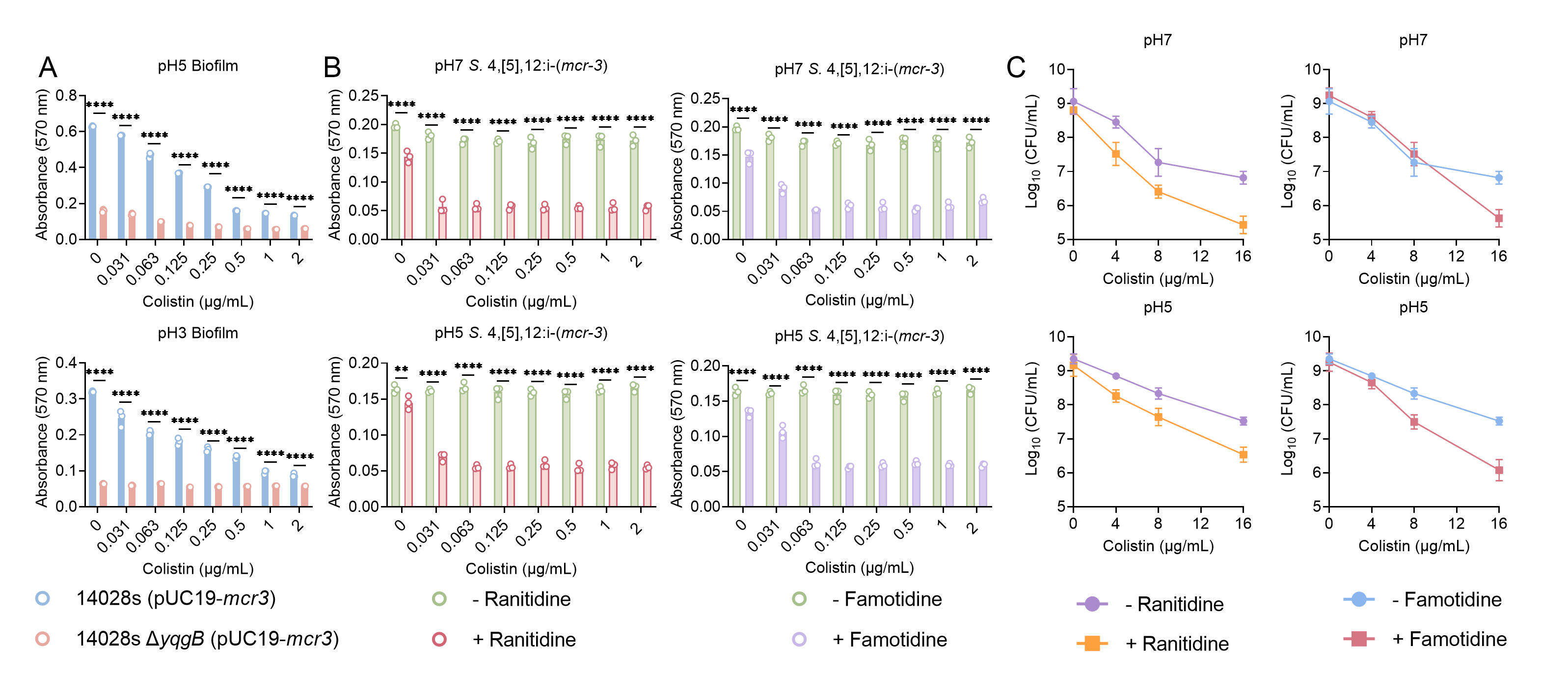


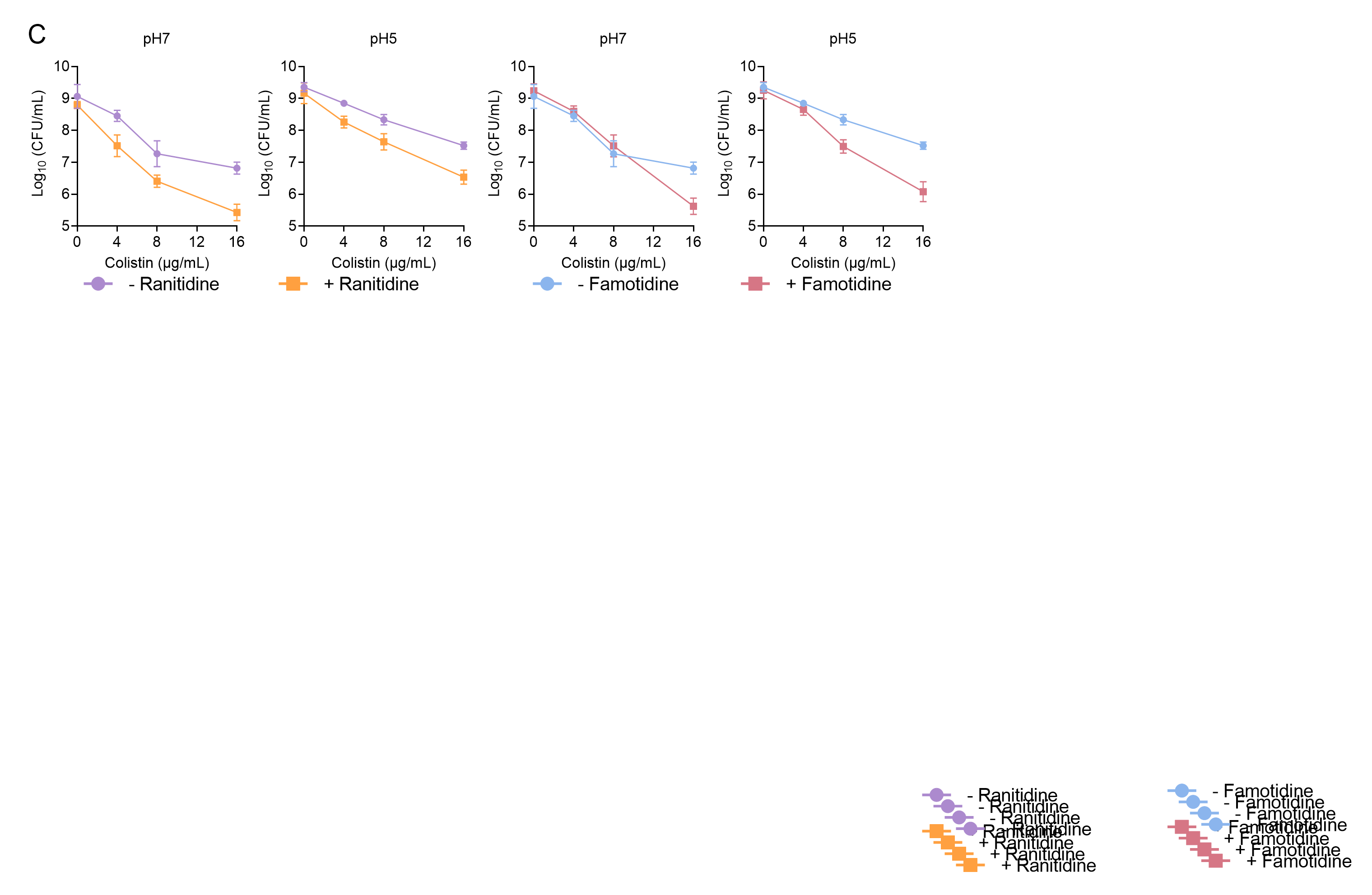


**Figure S9. Ranitidine/famotidine enhance the anti-biofilm activity of colistin under neutral and weakly acidic environments.**

**(A)** The deletion of *yqgB* gene inhibits the biofilm formation in *mcr*-positive bacteria under acidic environments. **(B)** Supplementing with ranitidine or famotidine enhances the inhibitory effect of colistin on biofilm formation of *S*. 4,[5],12:i- (*mcr-3*). **(C)** The addition of ranitidine or famotidine promoted the eradication of colistin against the established *S*. 4,[5],12:i- (*mcr-3*) biofilms. Data were presented as mean ± SD from three biological replicates, and significance was determined by two-way ANOVA (***P* < 0.01, *****P* < 0.0001).


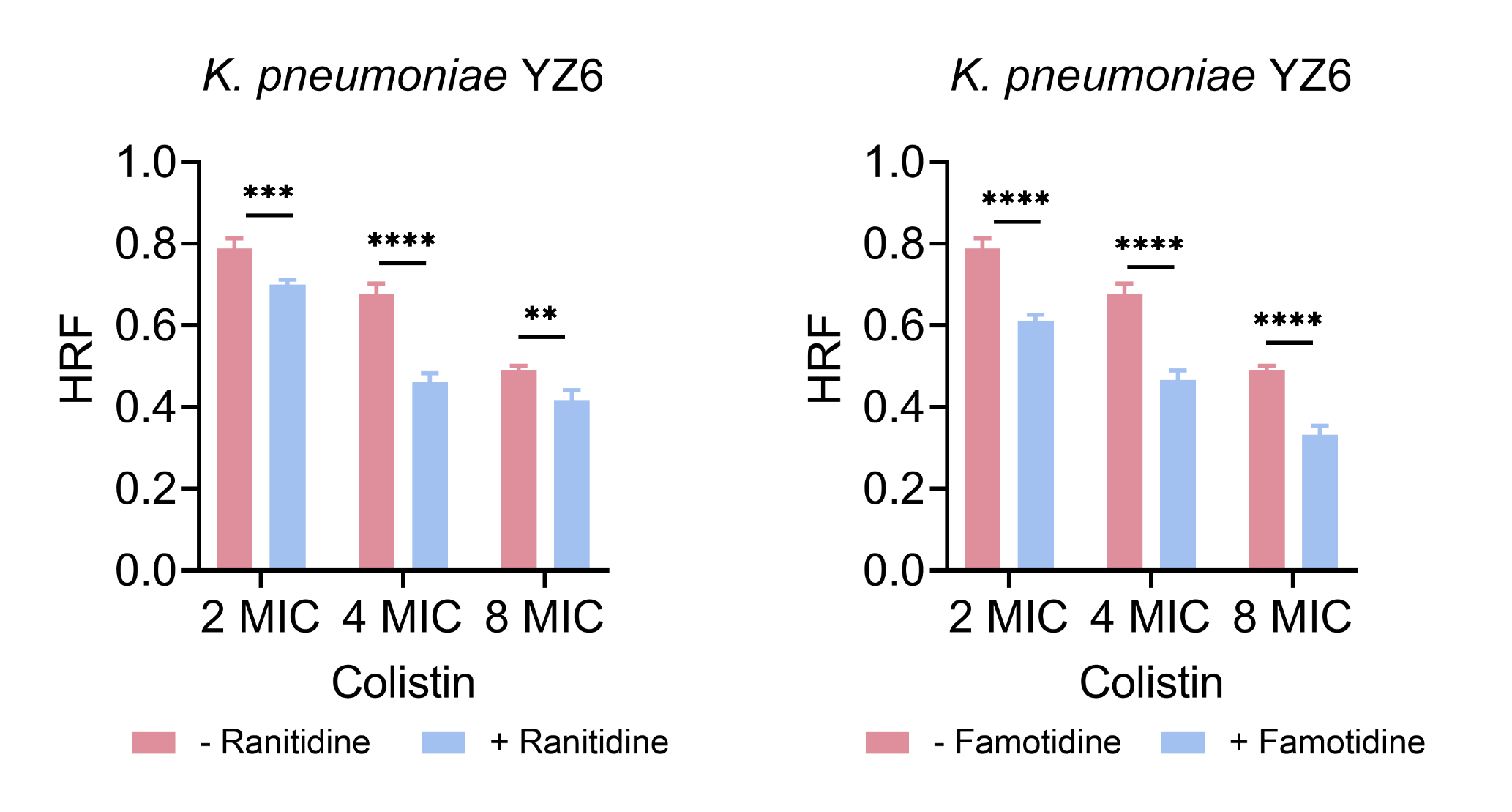


**Figure S10. Assessment of heteroresistance frequency in strain *K. pneumoniae* YZ6 following the addition of ranitidine or famotidine.** Data were presented as mean ± SD from three biological replicates, and significance was determined by two-way ANOVA (***P* < 0.01, ****P* < 0.001, *****P* < 0.0001).

**
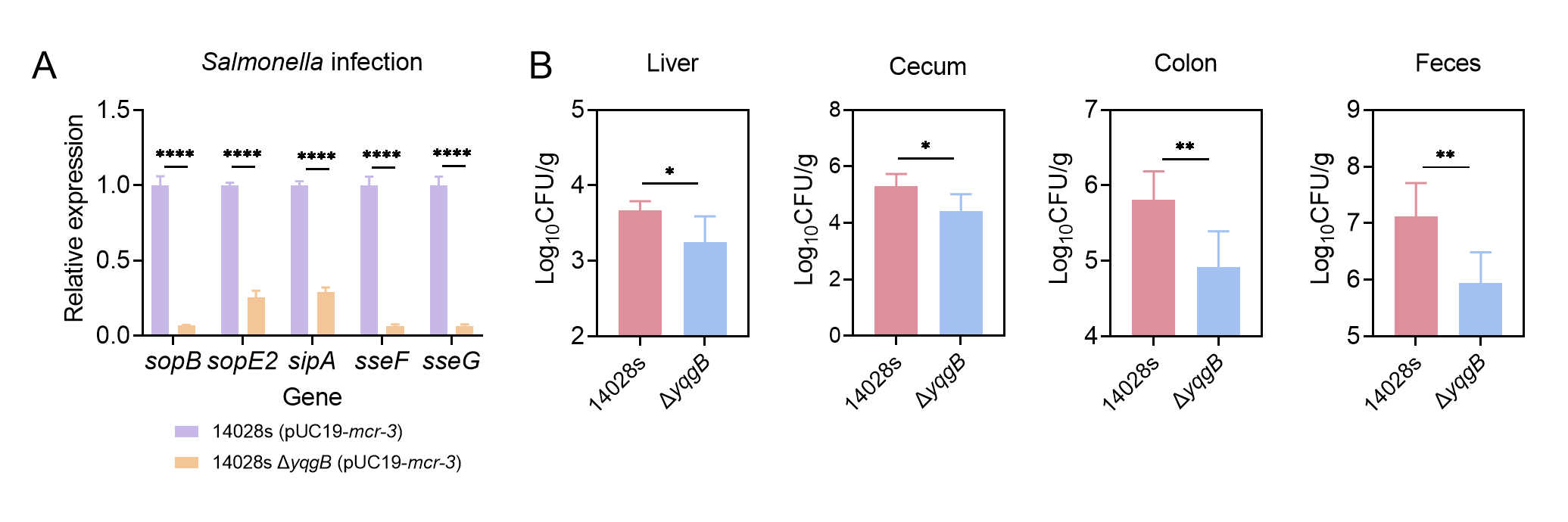
**

**Figure S11. The deletion of *yqgB* reduces the virulence of bacteria.**

**(A)** mRNA expression of *Salmonella* infection-related genes in the original strain and *yqgB-*deficient strain. Data were presented as mean ± SD from three biological replicates, and significance was determined by two-way ANOVA (*****P* < 0.0001). **(B)** Bacterial loads in the liver, cecum, colon, and feces of mice infected with the original strain and *yqgB* deficient strain 24 h after infection. Data were presented as mean ± SD from six biological replicates**,** and significance was determined by *t*-test (**P* < 0.05*, **P* < 0.01).


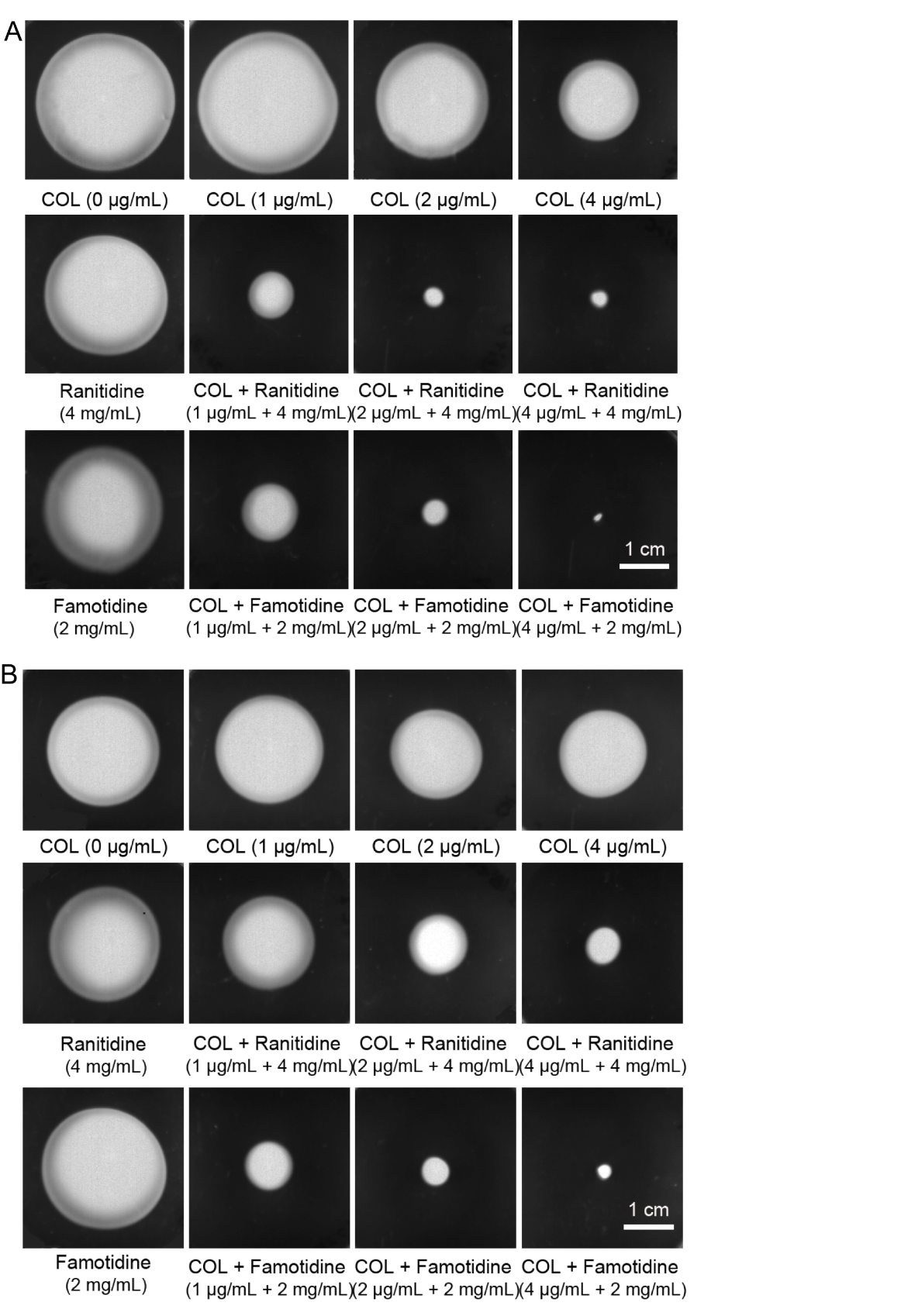


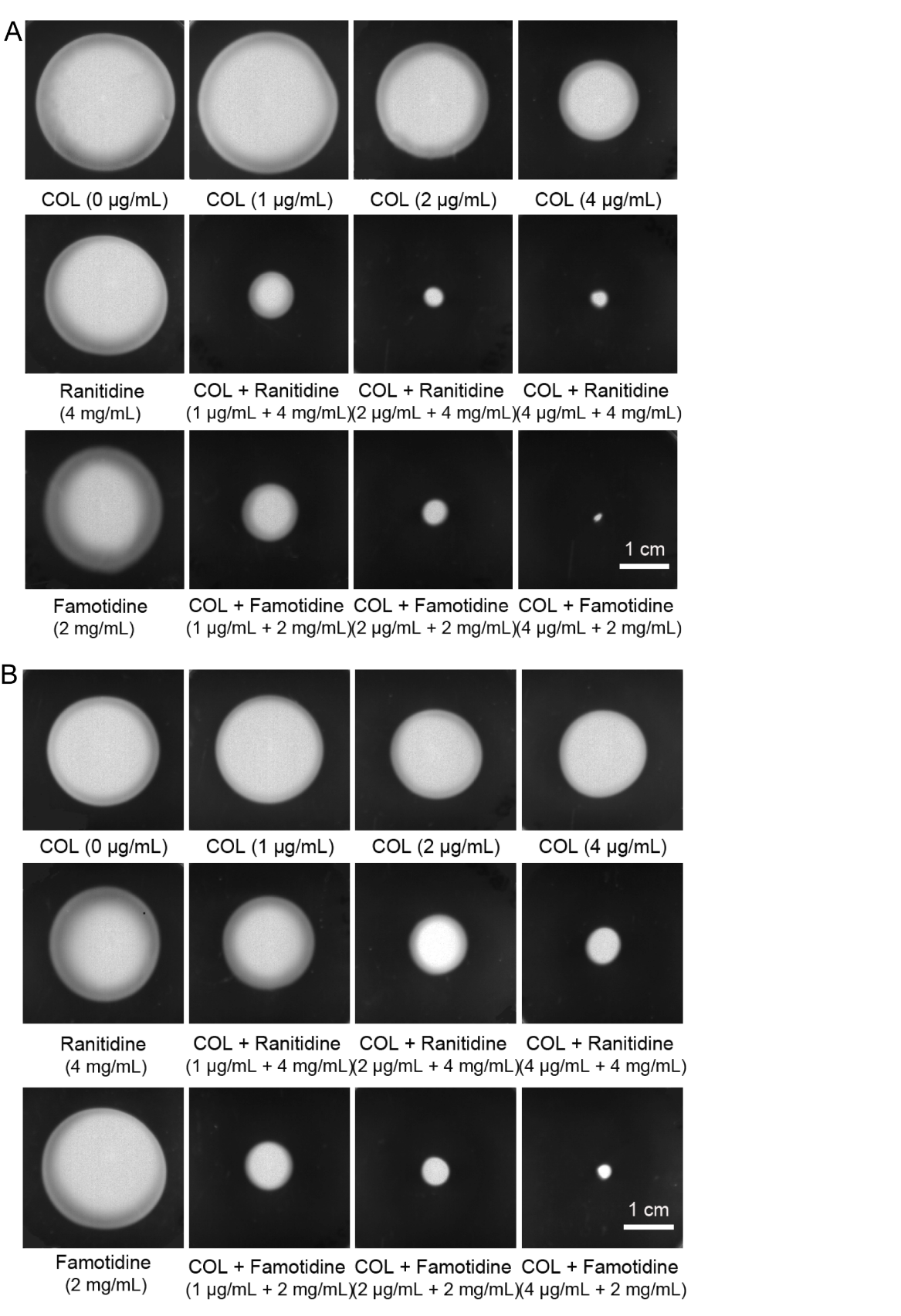


**Figure S12. The effect of colistin combined with ranitidine/famotidine on the swimming motility of *S*. 4,[5],12:i- (*mcr-3*) under neutral (A) and weakly acidic (B) conditions.**


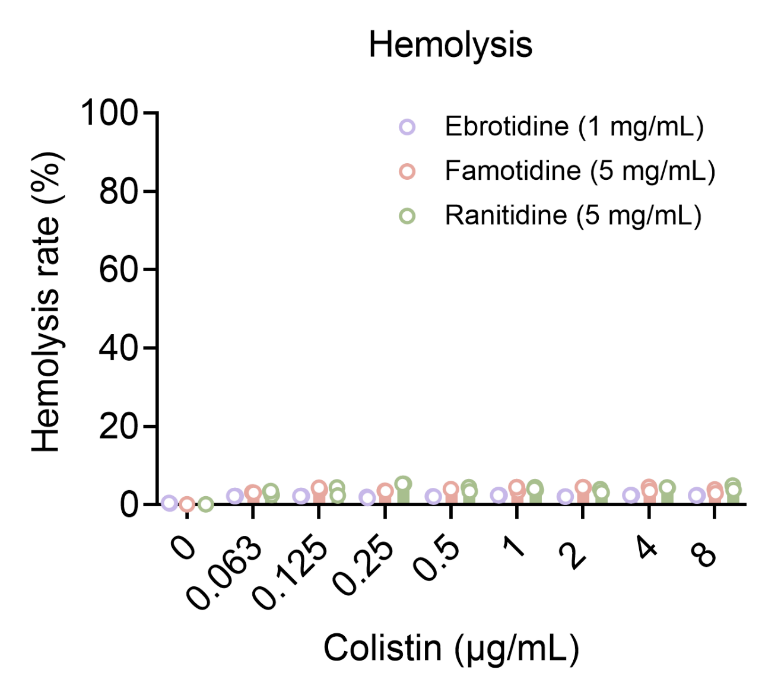


**Figure S13. Safety evaluation of ebrotidine/ranitidine/famotidine.**

Hemolytic activity of RBCs in the presence of increasing concentrations of colistin in combination with ebrotidine/ranitidine/famotidine. Data were presented as mean ± SD from three biological replicates.

**
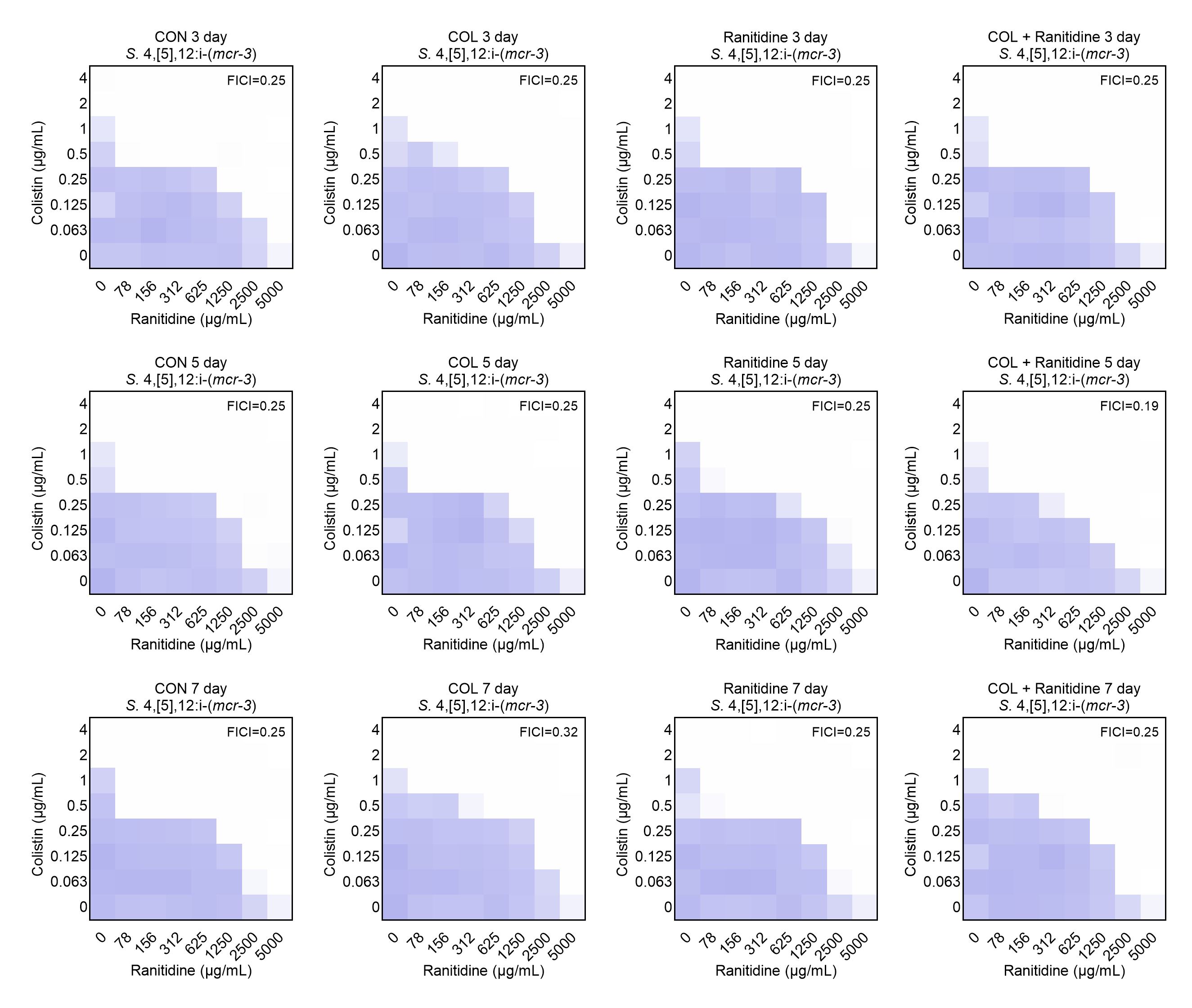
**

**Figure S14. The effect of different treatments on the development of bacterial resistance *in vivo*.**

On the 3rd, 5th, and 7th days of mouse infection treated with different drugs, bacteria were isolated and subjected to checkerboard assays between ranitidine and colistin.

**
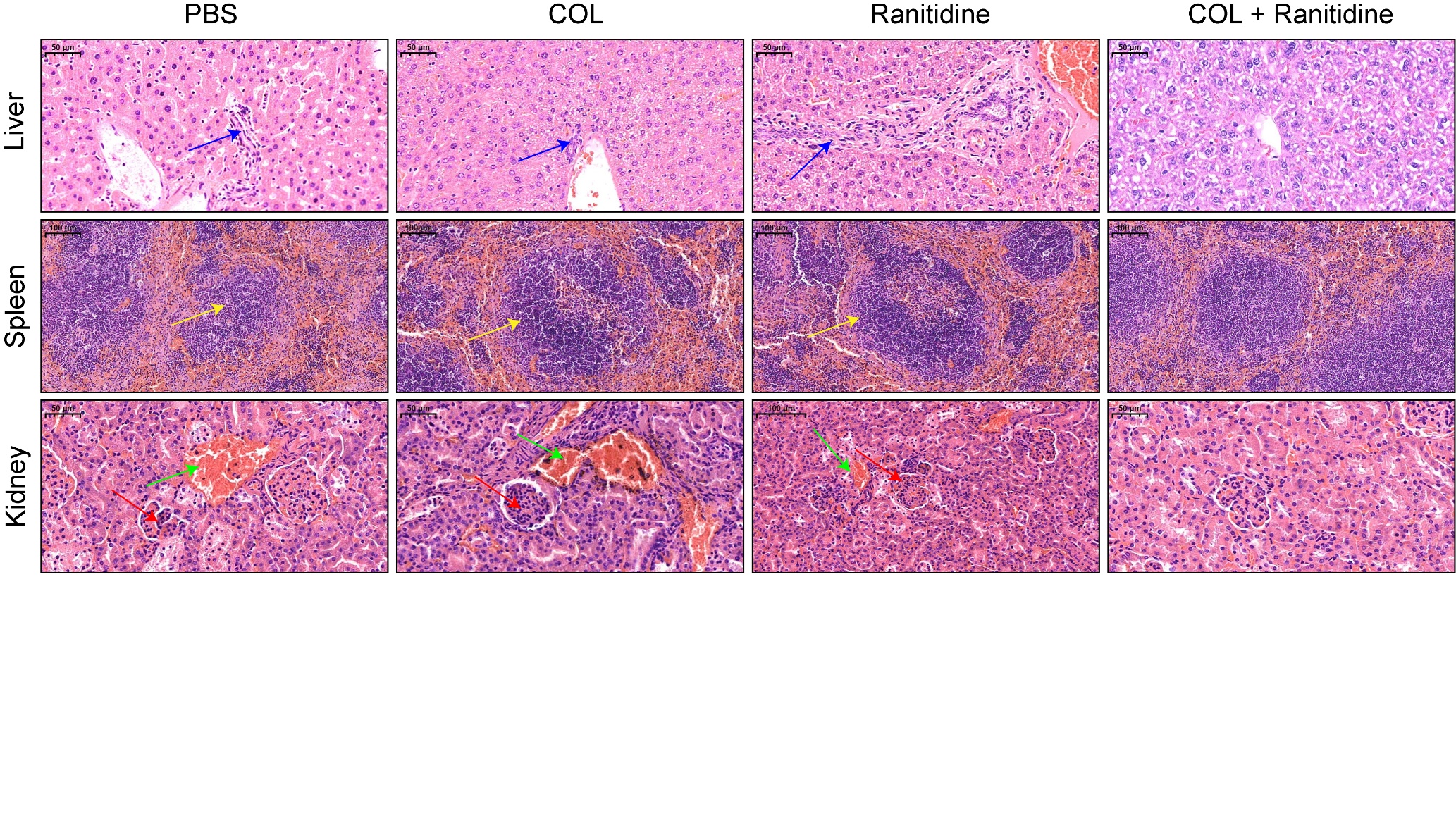
**

**Figure S15. Histopathological analysis of liver, spleen, and kidney after different treatments.**

The blue arrow in the liver indicates inflammatory cell infiltration. The yellow arrow in the spleen represents the disordered morphology and unclear boundaries in the white pulp of the spleen. The red arrow in the kidney indicates glomerular deformation, while the green arrow denotes tubular hemorrhage.

**Supplementary Tables**

**Table S1. MIC values of colistin on *S*. 4,[5],12:i- (*mcr-3*) after adding different compounds under neutral environment.**

| *S.* 4,[5],12:i- (*mcr-3*) | MIC (μg/mL) (pH=7) |
| --- | --- |
| Colistin | 2 |
| + Prazosin (100 μg/mL) | 2 |
| + Mycophenolate Mofetil (100 μg/mL) | 2 |
| + Chloroquine (100 μg/mL) | 2 |
| + Bevantolol hydrochloride (100 μg/mL) | 2 |
| + Acitazanolast (100 μg/mL) | 2 |
| + Voxelotor (100 μg/mL) | 2 |
| + Ebrotidine (100 μg/mL) | 0.25 |
| + Boldine (100 μg/mL**)** | 2 |
| + Naphazoline Hydrochloride (100 μg/mL) | 2 |
| + 3-Butylphthalide (100 μg/mL) | 1 |
| + 7-(B-hydroxypropyl) theophylline (100 μg/mL) | 2 |
| + Thiamine disulfide (100 μg/mL) | 2 |
| + Toloxatone (100 μg/mL) | 2 |

**Table S2. MIC values of colistin on *S*. 4,[5],12:i- (*mcr-3*) after adding different compounds under weakly acidic conditions.**

| *S.* 4,[5],12:i- (*mcr-3*) | MIC (μg/mL) (pH=5) |
| --- | --- |
| Colistin | 8 |
| + Prazosin (100 μg/mL) | 8 |
| + Mycophenolate Mofetil (100 μg/mL) | 8 |
| + Chloroquine (100 μg/mL) | 8 |
| + Bevantolol hydrochloride (100 μg/mL) | 8 |
| + Acitazanolast (100 μg/mL) | 8 |
| + Voxelotor (100 μg/mL) | 8 |
| + Ebrotidine (100 μg/mL) | 0.5 |
| + Boldine (100 μg/mL) | 8 |
| + Naphazoline Hydrochloride (100 μg/mL) | 8 |
| + 3-Butylphthalide (100 μg/mL) | 8 |
| + 7-(B-hydroxypropyl) theophylline (100 μg/mL) | 8 |
| + Thiamine disulfide (100 μg/mL) | 8 |
| + Toloxatone (100 μg/mL) | 8 |

**Table S3. Bacterial strains used in this study.**

| Strains | Sources |
| --- | --- |
| STm 14028s | ATCC |
| STm 14028s (pUC19-*mcr-3*) | In this study |
| STm 14028s ∆*yqgB* (pUC19-*mcr-3*) | In this study |
| STm 14028s ∆*pspB* (pUC19-*mcr-3*) | In this study |
| STm 14028s ∆*msgA* (pUC19-*mcr-3*) | In this study |
| *S*. 4,[5],12:i- (*mcr-3*) | ^[1]^ |
| *E. coli* B2 (*mcr-1*) | ^[2]^ |
| *E. coli* EC600 | ^[3]^ |
| *E. coli* LD67-1 (*mcr-1*) | ^[4]^ |
| *E. coli* CSZ4 (*mcr-1*)  *K. pneumoniae* YZ6 | ^[5]^  ^[3]^ |

**References**

[1] R. Li, K. Peng, W. Huang, X. Sun, Y. Huang, G. Lei, H. Lv, Z. Wang, X. Yang, *J. Infect.* **2022**, *85*, 702.

[2] Y. Liu, K. Yang, Y. Jia, Z. Wang, *ACS Infect. Dis.* **2019**, *5*, 2061.

[3] Y. Jia, B. Yang, J. Shi, D. Fang, Z. Wang, Y. Liu, *Pharmacol. Res.* **2022**, *175*, 105978.

[4] X. Lu, X. Xiao, Y. Liu, S. Huang, R. Li, Z. Wang, *mSphere* **2020**, *5*, e01221-20.

[5] J. Sun, L. X. Fang, Z. Wu, H. Deng, R. S. Yang, X. P. Li, S. M. Li, X. P. Liao, Y. Feng, Y. H. Liu, *Sci. Rep.* **2017**, *7*, 424.

**Table S4. Blood routine indexes of mice in acute toxicity test.**

| Indexes | Unit | Control | COL | COL + Ranitidine |
| --- | --- | --- | --- | --- |
| WBC | 10^9^ cells/L | 3.28 (0.8-6.8) | 3.09±0.82 | 4.18±0.90 |
| Lymph# | 10^9^ cells/L | 2.26 (0.7-5.7) | 2.19±0.86 | 3.4±0.63 |
| Mon# | 10^9^ cells/L | 0.03 (0-0.3) | 0.02±0.06 | 0.03±0.05 |
| Lymph% | % | 68.9 (55.8-90.6) | 70.9±2.4 | 81.3±1.6 |
| RBC | 10^12^ cells/L | 9.59 (6.39-9.42) | 9.56±0.24 | 8.97±0.53 |
| HGB | g/L | 146 (110-143) | 144±3.2 | 140±2.8 |
| MCV | fL | 63.4 (48.2-58.3) | 61.1±0.4 | 63.8±0.35 |
| MCH | pg | 15.2 (15.8-19) | 15.1±0.2 | 15.8±0.15 |
| PLT | 10^9^ cells/L | 819 (450-1590) | 811±24 | 791±27 |
| MPV | fL | 7.1 (3.8-6.0) | 7.0±0.1 | 6.8±0.15 |

**Table S5. Blood biochemical indexes of mice in acute toxicity test.**

| Indexes | Unit | Control | COL | COL + Ranitidine |
| --- | --- | --- | --- | --- |
| ALT | U/L | 26 (10-100) | 22±4.5 | 28±6.2 |
| AST | U/L | 103 (0-50) | 102±0.6 | 100±0.8 |
| AST/ALT |  | - | 4.64±0.2 | 3.57±0.1 |
| ALP | U/L | 60 (23-21) | 60±2 | 56±4.5 |
| TP | g/L | 62.1 (52-82) | 58.7±2.1 | 63.5±3.4 |
| ALB | g/L | 27 (23-40) | 28.5±1.4 | 28.3±2.2 |
| GLO | g/L | 31.7 (25-45) | 33.6±2.6 | 35.2±2.4 |
| A/G |  | 0.85 (1.2-2.3) | 0.85±0.4 | 0.8±0.38 |
| UREA | mmol/L | 4.7 (2.5-9.6) | 5.5±0.5 | 5.2±0.28 |
| UA | μmol/L | 111 (135-425) | 67±12 | 82±8 |
